# Genome-Wide Mutagenesis of *Xanthomonas axonopodis* pv. *citri* Reveals Novel Genetic Determinants and Regulation Mechanisms of Biofilm Formation

**DOI:** 10.1371/journal.pone.0021804

**Published:** 2011-07-05

**Authors:** Jinyun Li, Nian Wang

**Affiliations:** Department of Microbiology and Cell Science, Citrus Research and Education Center, University of Florida, Lake Alfred, Florida, United States of America; University of Vermont, United States of America

## Abstract

*Xanthomonas axonopodis* pv. *citri* (*Xac*) causes citrus canker disease, a major threat to citrus production worldwide. Accumulating evidence suggests that the formation of biofilms on citrus leaves plays an important role in the epiphytic survival of this pathogen prior to the development of canker disease. However, the process of *Xac* biofilm formation is poorly understood. Here, we report a genome-scale study of *Xac* biofilm formation in which we identified 92 genes, including 33 novel genes involved in biofilm formation and 7 previously characterized genes, *colR, fhaB, fliC, galU, gumD, wxacO, and rbfC*, known to be important for *Xac* biofilm formation. In addition, 52 other genes with defined or putative functions in biofilm formation were identified, even though they had not previously reported been to be associated with biofilm formation. The 92 genes were isolated from 292 biofilm-defective mutants following a screen of a transposon insertion library containing 22,000 *Xac* strain 306 mutants. Further analyses indicated that 16 of the novel genes are involved in the production of extracellular polysaccharide (EPS) and/or lipopolysaccharide (LPS), 7 genes are involved in signaling and regulatory pathways, and 5 genes have unknown roles in biofilm formation. Furthermore, two novel genes, *XAC0482*, encoding a haloacid dehalogenase-like phosphatase, and *XAC0494* (designated as *rbfS*), encoding a two-component sensor protein, were confirmed to be biofilm-related genes through complementation assays. Our data demonstrate that the formation of mature biofilm requires EPS, LPS, both flagellum-dependent and flagellum-independent cell motility, secreted proteins and extracellular DNA. Additionally, multiple signaling pathways are involved in *Xac* biofilm formation. This work is the first report on a genome-wide scale of the genetic processes of biofilm formation in plant pathogenic bacteria. The report provides significant new information about the genetic determinants and regulatory mechanism of biofilm formation.

## Introduction

Gram-negative plant pathogenic bacteria belonging to the genus *Xanthomonas* cause severe diseases in many economically important crop plants around the world and exhibit extremely high host-pathogen specificity [1]. *Xanthomonas* spp., including *Xanthomonas axonopodis* pv. *citri* (*Xac*) (syn. *Xanthomonas citri*, *Xanthomonas campestris* pv. *citri* or *Xanthomonas citri* subsp. *citri*) [2], [3], [4], have been used as model organisms for studying host-bacterium interactions [1], [5], [6], [7]. *Xac* causes citrus canker, a destructive disease in citrus, and affects most commercial varieties of citrus, limiting citrus production worldwide [8], [9]. *Xac* is typically spread by windblown rain and invades host plants directly through natural openings, such as stomata, and through wounds. The pathogen multiplies in intercellular spaces to cause canker disease [8]. Typical symptoms include raised corky lesions surrounded by a water or oil-soaked margin on leaves, stems, and fruits, inducing defoliation, twig dieback, general tree decline, blemished fruit, and premature fruit drop in severely infected trees [9]. Control is difficult in areas where the disease is already established and is based on the heavy use of copper-containing compounds. Recurrent and severe attacks of the disease are responsible for serious economic losses in citrus groves [9].

Early studies have shown that *Xac* forms biofilms on both abiotic and biotic surfaces [10], [11], [12]. Biofilms exhibit complex structures that involve groups of microcolonies attached by a bacterially produced matrix. Biofilms are of great medical, industrial, and agricultural interest because of their prevalence and general resistance to adverse conditions such as environmental stresses, host defense mechanisms, and antimicrobial treatment [13], [14]. In plant-bacteria interactions, biofilm formation has been reported to be implicated in the virulence of diverse bacterial pathogens [13]. The ability of the citrus canker pathogen to form biofilms enhances epiphytic persistence on host leaves, which plays an important role in the early stages of infection [12], [15].

Biofilm formation is a dynamic and complex process that generally includes the initial surface attachment of cells to the substratum, physiological changes within the organism, multiplication of the cells to form microcolonies, and eventually maturation of the biofilm [14]. Because of this complexity, knowledge regarding the process of biofilm formation and its regulation is limited. Thus, elucidation of the underlying genetic determinants and regulatory processes are prerequisites for further understanding the mechanism of biofilm formation [16]. The genome sequences of *Xac* and multiple close relatives have been sequenced [5], [17], which has facilitated research in this area. Tremendous progress has been made in identifying the genetic determinants and regulatory processes of *Xac*. These genes include the two-component signal transduction system (TCSTS) encoded by *colS/colR* [18], the filamentous hemagglutinin-like adhesin encoded by *fhaB* [10], the flagellin gene encoded by *fliC* [19], the UTP-glucose-1-phosphate uridylyltransferase encoded by *galU* [11], the xanthan EPS gene cluster encoded by *gumB* and *gumD* [12], [20], and the LPS biosynthesis associated genes encoded by *wxacO* and *rbfC* [15]. However, a comprehensive understanding of *Xac* biofilm formation has not yet been achieved because previous studies have focused only on individual genes and the specific genetic pathways responsible for biofilm formation. Transposon mutagenesis has been widely used for a global identification of biofilm-related genes in human bacterial pathogens [21], [22] and has the potential to identify novel *Xac* biofilm-related genes.

The goal of the present study was to advance our understanding of the underlying genetic determinants and regulatory processes of biofilm formation by the citrus canker bacterium *Xac*. An EZ-Tn5 library containing 22,000 *Xac* strain 306 mutants was used to study the process of biofilm formation. Screening for biofilm-defective mutants led to the identification of novel genetic determinants and an understanding of the regulatory mechanisms of *Xac* biofilm formation. Importantly, this work is the first report of the genetic process of biofilm formation by plant pathogenic bacteria on a genome-wide scale, and novel insights into the underlying mechanisms of biofilm formation revealed by this work hold the potential for exploitation to improve control strategies of citrus canker.

## Results

### Isolation and characterization of biofilm-defective mutants

**Figure 1:**
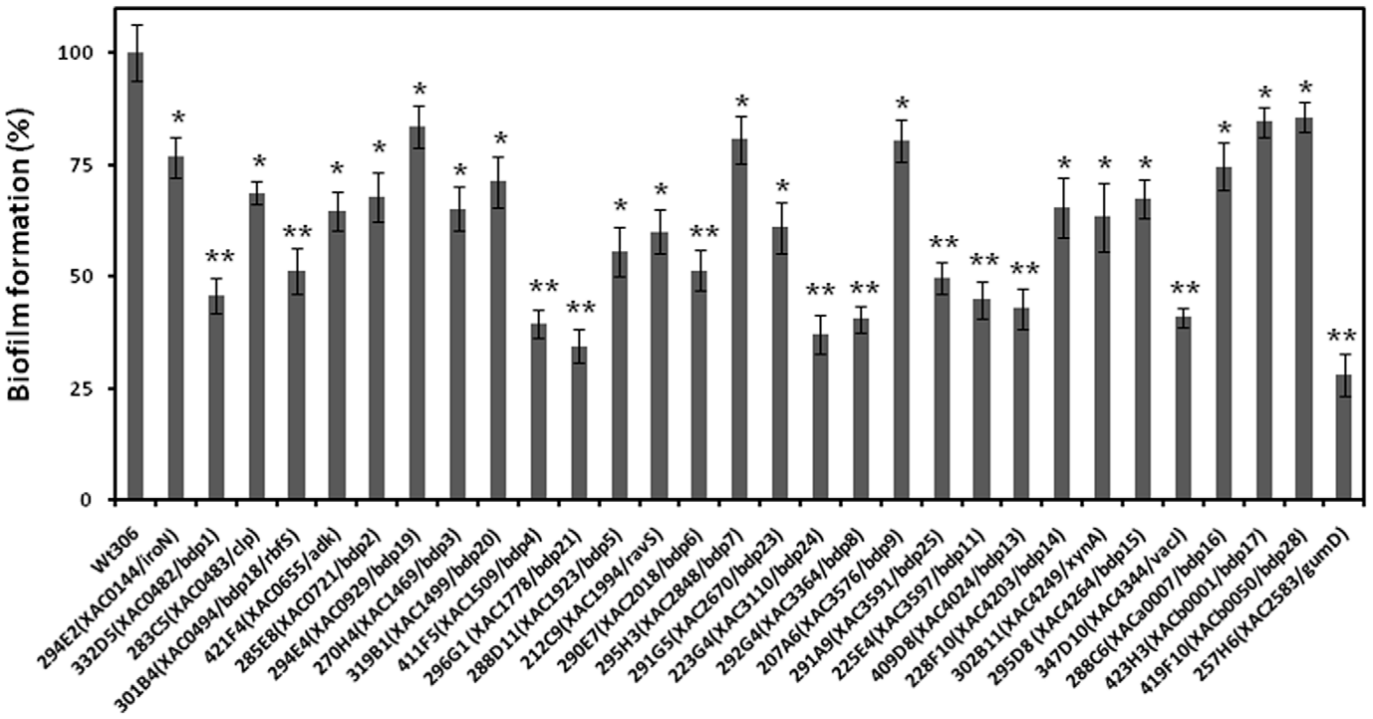
Quantitative biofilm assay in borosilicate glass tubes showing the ability to form biofilm by a proportion of the EZ-Tn5 mutants of *Xanthomonas axonopodis* pv. *citri* strain 306. The biofilm was determined using crystal violet staining coupled with measurements of absorbance at 590 nm (A_590_). The experiment was repeated three times with five replicates. Averages and standard errors from one of three representative experiments with similar results are shown. Wild-type strain 306 was set as equal to 100%. Statistical significance was tested by Student's *t-*test (* indicates significant differences from wild-type strain 306 at *P*<0.01; ** indicates significant differences from wild-type strain 306 at *P*<0.001).

To further understand citrus canker bacterium biofilm formation, an EZ-Tn5 mutant library containing 22,000 *Xac* strain 306 mutants [11] was screened for biofilm-defective mutants to identify genes involved either directly or indirectly in biofilm formation. During the initial screening, 327 mutants were identified as biofilm-defective mutants based on the reduced ability of the bacteria to adhere to the surface of the wells of polystyrene 96-well microtiter plates, as visualized by staining with crystal violet. Mutants that lacked a violet ring formation on the side of each well or a generalized staining of the well were scored as having a biofilm-defective phenotype. Following further confirmation of these phenotypes by quantitative biofilm tests in borosilicate glass tubes, the growth of each strain was assessed. After the elimination of mutants with inconsistent biofilm formation or general growth deficiencies, 292 mutants were confirmed as being biofilm-defective mutants (Fig. 1 and data not shown) and subjected to further investigation. All 292 mutants exhibited significant (*P*<0.01 by Student's t-test) and strong (from 20% to 80%) defects in biofilm formation compared to wild-type *Xac* strain 306 (Fig. 1 and data not shown). For convenience, these mutants were designated as *b*iofilm-*d*efective *p*henotype (bdp) mutants. These 292 bdp mutants represented 1.32% of the 22,000 mutants tested.

To test the EZ-Tn5 transposon copy number in each mutant, Southern blotting was performed with the kanamycin resistance gene as a probe. Southern hybridization analysis revealed a single band in 16 out of 17 bdp randomly selected EZ-Tn5 insertion mutants in novel genes (Table 1 and see below), but no bands were detected in the wild-type *Xac* strain 306 (Supporting Information Figure S1); a double insertion was found in only one mutant, suggesting a high ratio of single transposon insertions in the genome as described by the EZ-Tn5™ <R6Kγori/KAN-2>Tnp Transposome™ Kit (Epicentre, Madison, WI).

**Table 1:** Novel biofilm-related genes identified from *Xanthomonas axonopodis* pv. *citri* strain 306 in this study.

Locus ofEZ-Tn5 tagged^a^	Genename	Gene product or domain similarity^b^	Mutantstrains		Phenotypes^c^	
				EPS production(%)	Swimming motility(%)	Swarming motility(%)
			WT306^d^	100±4.2	100±4.3	100±1.2
*XAC0482*	*bdp1*	hypothetical protein, similar to haloacid dehalogenase-like hydrolase/phosphatase	332D5	25.3±1.3*	67.6±2.1*	48.1±1.2*
*XAC0721*	*bdp2*	hypothetical protein, predicted endonuclease/exonuclease/phosphatase family	285E8	68.8±1.9*	89.1±2.7*	92.8±2.8
*XAC1469*	*bdp3*	hypothetical protein, similar to TatD-related DNase	270H4	78.8±2.6*	93.3±3.9	94.1±2.3
*XAC1509*	*bdp4*	hypothetical protein	411F5	65.1±2.1*	92.1±2.3	62.7±1.1*
*XAC1923*	*bdp5*	hypothetical protein	288D11	92.8±3.8	92.7±3.6	92.4±2.2
*XAC2018*	*bdp6*	hypothetical protein, predicted transcriptional regulator containing an XRE-like HTH domain	290E7	103.9±4.6	79.7±1.6*	75.3±1.1*
*XAC2848*	*bdp7*	hypothetical protein, similar to the lipocalin-like protein	295H3	77.6±3.3*	92.4±3.3	81.9±0.9*
*XAC3364*	*bdp8*	hypothetical protein, similar to acetyl-CoA hydrolase	292G4	93.6±3.2	67.9±2.1*	58.3±0.8*
*XAC3576*	*bdp9*	hypothetical protein, putative carbohydrate biosynthesis protein, contains a Pfam_Rgpf domain	207A6	92.7±3.2	93.8±2.7	93.1±2.2
*XAC3597*	*bdp11*	hypothetical protein, similar to phytanoyl-CoA dioxygenase, Phyh superfamily	225E4	92.9±4.2	84.8±4.1*	98.8±2.2
*XAC4024*	*bdp13*	hypothetical protein, similar to outer membrane protein	409D8	94.1±3.9	97.1±4.5	101.3±2.4
*XAC4203*	*bdp14*	hypothetical protein, similar to putative pathogenicity protein	228F10	56.6±2.7*	92.4±4.8	80.3±1.9*
*XAC4264*	*bdp15*	hypothetical protein, similar to sucrose isomerase	295D8	53.9±2.2*	93.3±4.3	89.4±2.1*
*XACa0007*	*bdp16*	hypothetical protein, similar to protein kinase C	288C6	77.9±2.7*	94.6±5.1	93.7±2.5
*XACb0001*	*bdp17*	hypothetical protein, similar to Radical SAM domain protein	423H3	92.4±3.1	95.3±3.4	102.5±1.4
*XAC0144*	*iroN*	TonB-dependent outer membrane receptor	294E2	98.3±3.8	98.1±3.4	96.9±2.1
*XAC0494*	*bdp18/rbfS*	two-component system sensor protein, with a histidine kinase domain, a cheY receiver domain and a histidine phosphotransfer domain	301B4	79.1±3.1*	79.6±2.9*	75.3±2.1*
*XAC0929*	*bdp19*	extracellular protease	294E4	95.3±4.1	95.6±4.8	94.6±2.7
*XAC1499*	*bdp20*	transcriptional regulator, XRE family	319B1	69.9±1.9*	94.4±3.7	84.8±1.6*
*XAC1778*	*bdp21*	sensor kinase, MASE1-containing protein, similar to sugar transporter component	296G1	47.9±1.8*	54.5±1.7*	61.4±0.8*
*XAC2670*	*bdp23*	alginate biosynthesis protein, similar to the two-component system sensor protein	291G5	94.7±3.8	70.5±2.6*	73.4±1.1*
*XAC3110*	*bdp24*	glycosyltransferase, glycosyl transferase family 2	223G4	37.0±1.3*	65.5±2.2*	46.2±0.7*
*XAC3591*	*bdp25*	short chain dehydrogenase	291A9	94.2±3.6	85.6±3.6*	94.6±1.7
*XAC4249*	*xynA*	endo-1,4-beta-xylanase	302B1	71.5±3.1*	92.6±4.7	96.2±1.7
*XAC4344*	*vacJ*	lipoprotein	347D1	94.5±3.3	97.9±3.3	100.8±2.5
*XACb0050*	*bdp28*	ISxac2 transposase	419F1	80.7±2.2*	95.7±2.8	98.1±1.5

a Open reading frame numbering (ordered sequence tag) from the strain 306 genome.

b Based on BLASTP search and SMART analyses.

c Each test, with three replicates, was repeated three times with similar results. Means and standard errors of three replicates from one representative experiment are shown. Data with ‘*’ in the same column are significantly different from wild-type strain 306 at *P* <0.01 (Student's t-test). Wild-type strain 306 was set as equal to 100%.

d Wild-type strain 306.

### Genomic distribution of transposon-tagged biofilm related genes

The precise EZ-Tn5 insertion sites of the bdp mutants were determined by sequencing analysis based on the random amplification of transposon ends polymerase chain reactions (RATE-PCR) [23]. Sequences flanking transposon insertions were identified by a homology search of the entire *Xac* strain 306 genome sequence (GenBank accession no. AE008923) using the BLASTN search algorithm. To verify the accuracy of transposon insertions in the sequences, PCR amplification was performed using primers designed from the sequences flanking the ORF of target genes. The size of each PCR product from the mutants was increased by the insertion of a transposon (1980 bp), indicating that the transposons were precisely inserted into target genes in the *Xac* genome (data not shown).

In some cases (approximately 40% of the identified mutants), two or more independently derived mutants mapping to the same gene coding region were found, but at different locations. In these cases, only a single mutant from each gene was chosen for further analyses. In total, ninety-two distinct genes and four intergenic regions were identified (Supporting Information Table S1), representing 2.1% of the 4427 predicted open reading frames (ORFs) in the chromosomal genome (4312 ORFs) and in plasmids (115 ORFs) [5]. Ninety-three insertions occurred in the chromosome of *Xac* strain 306, two insertions were on the pXAC66 plasmid, and one insertion was on the pXAC33 plasmid. The transposons were widely inserted in diverse *Xac* genes, suggesting genome-wide insertions, and transposon target genes were randomly distributed throughout the *Xac* strain 306 genome (Supporting Information Figure S2).

Transposon insertion may cause a polar effect that affects the normal transcription of downstream genes within the same operon. The availability of *Xac* strain 306 genomic sequences made it possible to predict polar effects in the obtained mutants. Based on the results of transcription-unit prediction by BioCyc [24] and the insertion direction of the transposon, all biofilm-formation-related genes identified in this study could be divided into two groups. One contains 66 genes that are organized as a monocistron or at the 3′ end of operons, or at the 5′ end of operons and transposon insertions with the same transcription direction as the operons. In this group of 66 genes, transposon insertions may not cause polar effects (Supporting Information Table S1). The other group of biofilm-formation-related genes contained 26 genes with insertions that at the 5′ end of operons but with the insertion occurring in the reverse transcriptional direction from the operons. Transposon integration in these genes may lead to polar effects on downstream gene expression that complicate phenotypic characterization.

### Functional classification of biofilm-formation-related genes

As suggested by the Kyoto Encyclopedia of Genes and Genomes (KEGG) pathway[25], the 92 biofilm-formation-related genes are involved in carbohydrate, amino acid, nucleotide, or energy metabolism; bacterial chemotaxis and motility; flagellum or pilus assembly; DNA replication and repair; transcription; membrane transport; signal transduction; signaling molecule biosynthesis and interaction; not well characterized and unknown functions (Supporting Information Table S1). Interestingly, 17 out of the 92 genes were previously unknown genes encoding hypothetical proteins whose functions have yet to be assigned. There were 16 other genes assigned putative functions, including 11 without gene names that belonged to the “not well characterized” gene family based on the KEGG pathway [25]. Therefore, these 28 genes, which included the 17 hypothetical genes and the 11 genes without gene names, were designated as *bdp* genes for their biofilm-defective phenotype (Supporting Information Table S1). Among the 64 other genes, 7 (*colR*, *fhaB*, *fliC*, *galU*, *gumD*, *wxacO,* and *rbfC*) were previously characterized as being important for *Xac* biofilm formation, and the 57 other genes were assigned putative functions and may be involved in biofilm formation (Supporting Information Table S1).

### Transposon insertions in known loci related to biofilm formation

As mentioned above, the 92 biofilm-formation-related genes identified in this study contain both genes that were known to be linked to biofilm formation and genes that were not known to be associated with biofilm. In the category of known loci, a subset of 7 genes (Supporting Information Table S1), i.e., *colR* [18], *fhaB* [10], *fliC* [19], *galU* [11], *gumD* [20], *wxacO,* and *rbfC* [15], were previously characterized to be important for *Xac* biofilm formation. In addition to the 7 genes mentioned above, we uncovered 52 genes with defined or putative functions involved in biofilm formation, although they had not been previously reported to be involved in *Xac* biofilm formation (Supporting Information Table S1; Supporting Information Figure S3). These genes included thirteen flagellum biosynthesis genes (*fleN, flgABFKL, flhB, fliCFMQR*); six bacterial chemotaxis and motility genes (*cheA, cheY, mcpA, motB, motD* and *tsr*); one pilus biosynthesis protein (*pilB)*; five gum genes (*gumCEFJK*); three LPS biosynthesis genes (*ipsJ*, *rmlA* and *rmlB*); multiple enzymes involved in carbohydrate, amino acid, nucleotide, or energy metabolisms (*bioB, ilvE, ldp, metB, nuoM, rpfF, sahH, thiE, thiG, trpE, ugd, xanA,* and *xanB*); DNA replication and repair (*mrdB* and *nrdF*); signal transduction and transcriptional regulators (*clp, opsX, rfpC* and *rpoN*); and transporters (*iroN, msbA, sbp, wzm* and *wzt*). Inactivation of orthologs of these genes led to biofilm-defective phenotypes in a variety of bacteria [14].

### Novel genes associated with EPS production contribute to biofilm formation

In addition to the known loci associated previously with biofilm formation, we found 33 genes that were either hypothetical genes or not well-characterized genes assigned putative functions based on homology analyses, and of these, 28 genes were designated as *bdp* genes (see above). Notably, 26 of the 28 *bdp* genes contained transposon insertions that did not result in polar effects (Supporting Information Table S1). As an initial step in investigating the roles of these novel genes in biofilm formation, we first characterized genes that were potentially associated with polysaccharide production because EPS is the major structural constituent of many bacterial biofilms [14], [16].

Our data revealed that a total of 13 *bdp* gene mutants, including 332D5 (*XAC0482/bdp1*), 301B4 (*XAC0494/bdp18/rbfS*), 285E8 (*XAC0721*/*bdp2*), 270H4 (*XAC1469*/*bdp3*), 319B1 (*XAC1499*/*bdp20*), 411F5 (*XAC1509/bdp4*), 296G1 (*XAC1778/bdp21*), 295H3 (*XAC2848/bdp7*), 223G4 (*XAC3110/bdp24*), 228F10 (*XAC4203/bdp14*), 295D8 (*XAC4264/bdp15*), 288C6 (*XACa0007/bdp16*), and 419F10 (*XACb0050/bdp28*), were significantly reduced in total EPS production compared to wild-type strain 306 (*P*<0.01, Student's t-test). The 13 other mutants that were tested produced similar levels of EPS to those produced by wild-type strain 306 (Table 1).

Based on the putative functions of their corresponding gene products (Table 1), these EPS-related genes could be classified into three groups. The first group consisted of those whose corresponding gene products were probably enzymes directly involved in carbohydrate biosynthesis and/or metabolism, which included the *XAC0482/bdp1, XAC3110/bdp24*, and *XAC4264/bdp15* genes. The protein encoded by *XAC0482/bdp1* contains a haloacid dehalogenase (HAD)-like hydrolase domain and shares significant similarity (>95% amino acid identity) with members of an HAD-like hydrolase/phosphatase family that is conserved in most *Xanthomonas* species. Proteins of this family have been found in various bacteria, but most remain uncharacterized [26]. The putative XAC3110 protein contains a glycosyl transferase family 2 domain and a UDP-Glycosyltransferase/glycogen phosphorylase domain. The glycosyl transferase family 2 plays a general role in polysaccharide biosynthesis [27]. The *XAC4264/bdp15* gene encodes a 279 amino acid hypothetical protein with 40% similarity in amino acid sequence to the PalI sucrose isomerase from *Erwinia tasmaniensis* Et1/99, a non-pathogenic epiphytic plant bacterium [28]. Sucrose isomerase has been reported in a wide range of bacterial species, and in addition to catalyzing the isomerization of sucrose to isomaltulose, it also produces another sucrose isomer, trehalose, and glucose and fructose byproducts [29]. Earlier studies suggested that the XAC4264 protein interacts with VirD4, a component of the type IV secretion system (T4SS), and XAC4264 may act as a cofactor or substrate of the *Xanthomonas* T4SS [30]. Our data suggest that the XAC4264 protein is involved in EPS production (Table 1); however, whether it encodes a sucrose isomerase remains to be elucidated.

A second group of EPS-related genes contained five genes, *XAC0494/bdp18/rbfS*, *XAC0721*/*bdp2, XAC1499*/*bdp20*, *XAC1778/bdp21* and *XACa0007/bdp16*, whose corresponding gene products are potentially associated with signaling and regulation pathways, which are described below (Table 1).

A third group of EPS-related genes were those whose corresponding gene products could not be attributed to the above two groups. This group included the *XAC1469*/*bdp3*, *XAC1509/bdp4*, *XAC2848/bdp7*, *XAC4203/bdp14*, and *XACb0050/bdp28* genes. The putative XAC1469/Bdp3 protein is a hypothetical protein with a TatD_DNase domain. The TatD-related DNase family is a family of Mg-dependent DNases that participate in DNA replication, recombination and repair [31]. The *XAC1509/bdp4* gene was annotated as a hypothetical protein without any conserved domains. The closest homolog (80% amino acid identity) to XAC1509 was found in *X. campestris* pv. *campestris* strain B100 with a protein of unknown function. The putative 363 amino acid XAC2848 protein contains a predicted DUF3616 domain (Pfam entry PF12275). This family of proteins is mainly found in bacteria with unknown functions. The XAC4203 protein is a 1284 amino acid hypothetical protein that has a predicted signal peptide domain and a DUF0496 domain (Pfam entry PF04357) with an uncharacterized function at its C-terminal. Interestingly, the XAC4203 homolog of PXO_03725 in *X. oryzae* pv. *oryzae* strain PXO99A was annotated as a pathogenicity protein [7]. However, the role of PXO_03725 in pathogenesis remains unknown. The deduced XACb0050 protein is an ISxac2 transposase [5]. Bacterial transposase has been reported to be associated with biofilm formation. For example, the transposase encoded by IS256 in *Staphylococcus epidermidis* has the capacity to influence biofilm formation, either by insertion into regulatory genes or by modulating biofilm gene expression [32].

### Novel genes involved in LPS biosynthesis contribute to biofilm formation

**Figure 2:**
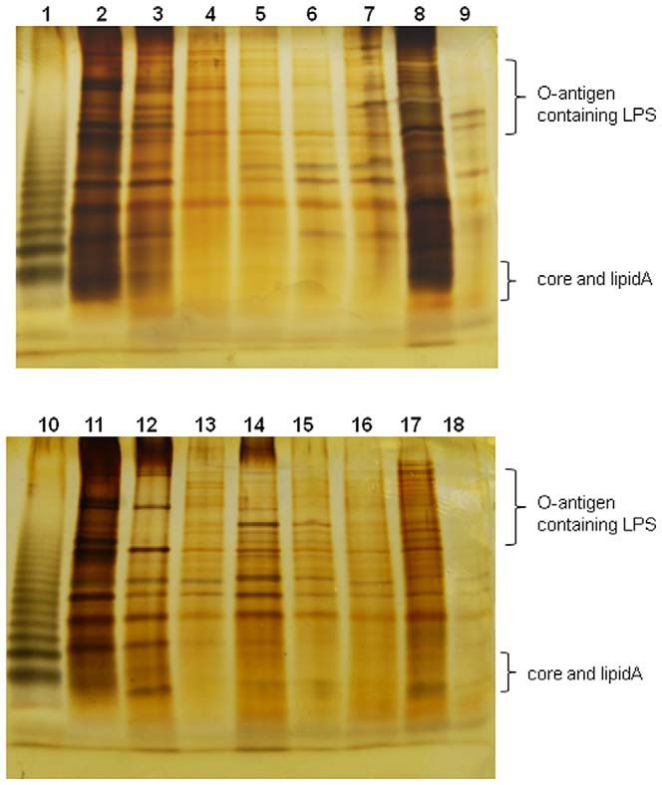
SDS-PAGE analysis of LPS produced by *Xanthomonas axonopodis* pv. *citri* strain 306 and its EZ-Tn5 insertion mutants. LPS samples were extracted and subjected to SDS-PAGE analysis and visualized by silver staining. Lanes: 1, 10: LPS standard from *Salmonella enteritidis* serovar Typhimurium (10 µg, Sigma); 2, 11: wild-type strain 306; 3: 332D5 (*XAC0482/bdp1*); 4: 285E8 (*XAC0721/bdp2*); 5: 270H4 (*XAC1469/bdp3*); 6: 288D11 (*XAC1923/bdp5*); 7: 290E7 (*XAC2018/bdp6*); 8: 295H3 (*XAC2848/bdp7*); 9; 292G4(*XAC3364/bdp8*); 12: 223G4 (*XAC3110/bdp24*); 13: 291G5(*XAC2670/bdp23*); 14: 207A6 (*XAC3576/bdp9*); 15: 228F10 (*XAC4203/bdp14*); 16: 301B4 (*XAC0494/bdp18/rbfS*); 17: 319B1 (*XAC1499/bdp20*); and 18: 296G1 (*XAC1778/bdp21*).

Because LPS has been demonstrated to be directly involved in biofilm formation in a number of bacteria, including *Escherichia coli* [22] and *Pseudomonas aeruginosa* [33], we were interested in determining whether the above-mentioned 26 *bdp* genes were associated with LPS biosynthesis in *Xac*. Sodium dodecyl sulfate polyacrylamide gel electrophoresis (SDS-PAGE) analysis revealed that LPS patterns, especially O-antigen profiles, were altered in seven *bdp* mutants, including 285E8 (*XAC0721*/*bdp2*), 296G1 (*XAC1778/bdp21*), 295H3 (*XAC2848/bdp7*), 23G4 (*XAC3110/bdp24*), 292G4 (*XAC3364/bdp8*), 207A6 (*XAC3576/bdp9)*, and 228F10 (*XAC4203/bdp14*), when compared with wild-type strain 306 (Fig. 2).

Interestingly, five mutants, including 285E8 (*XAC0721*/*bdp2*), 296G1 (*XAC1778/bdp21*), 295H3 (*XAC2848/bdp7*), 223G4 (*XAC3110/bdp24*), and 228F10 (*XAC4203/bdp14*), were also altered for EPS production (see above). Thus, these genes may play a role in the biosynthesis or transportation of certain precursors of EPS and LPS.

The product of the *XAC3364/bdp8* locus is a putative acetyl-CoA hydrolase. In some bacteria, e.g. *Pseudomonas reinekei,* the acetyl-CoA hydrolase is able to catalyze the hydrolysis of acetyl-CoA, glutaryl-CoA, and 3-butenoyl-CoA and is presumably involved in both central metabolism and energy metabolism [34], [35]. However, it is unknown how acetyl-CoA hydrolase is involved in LPS production or how it contributes to biofilm formation in bacteria.

The *XAC3576/bdp9* gene encodes a hypothetical protein containing a signal peptide and a transmembrane segment at the N-terminal region in addition to an RgpF (rhamnose-glucose polysaccharide assembly protein F) domain (Table 1). Members of the RgpF family have been suggested to be involved in the assembly of the LPS O-polysaccharides [36], [37].

### Flagellum-independent motility plays an important role in biofilm formation

Given that both flagellum-dependent and flagellum-independent motility are required for the formation of mature biofilm by a variety of bacteria, including *Xac* [19; for review see 14], we attempted to determine whether the 26 *bdp* genes are involved in *Xac* cell motility. A significant reduction (*P*<0.01, Student's t-test) in both swimming and swarming motility was observed in the following seven mutants: 332D5 (*XAC0482/bdp1*), 301B4 (*XAC0494/bdp18/rbfS*), 296G1 (*XAC1778/bdp21*), 290E7 (*XAC2018/bdp6*), 291G5 (*XAC2670/bdp23*), 223G4 (*XAC3110/bdp24*), and 292G4 (*XAC3364/bdp8*) (Table 1). In addition, the 285E8 (*XAC0721*/*bdp2*), 291A9 (*XAC3591/bdp25*) and 225E4 (*XAC3597/bdp11*) mutants showed decreased swimming motility, whereas the 411F5 (*XAC1509/bdp4*), 319B1 (*XAC1499/bdp20*), 228F10 (*XAC4203/bdp14*), and 295D8 (*XAC4264/bdp15*) mutants showed differences in swarming motility. The other *bdp* mutants had a similar motility to the wild-type 306 strain (Table 1).

The motility-impaired mutants could be grouped into two categories: 1) mutations potentially associated with signaling and regulation pathways, including the 301B4 (*XAC0494/bdp18/rbfS*), 285E8 (*XAC0721*/*bdp2*), 319B1 (*XAC1499/bdp20*), 296G1 (*XAC1778/bdp21*), 290E7 (*XAC2018/bdp6*), and 291G5 (*XAC2670/bdp23*) mutants, and 2) mutants that were also impaired in EPS and/or LPS production. This group included the 332D5 (*XAC0482/bdp1*), 411F5 (*XAC1509/bdp4*), 223G4 (*XAC3110/bdp24*), 292G4 (*XAC3364/bdp8*), 291A9 (*XAC3591/bdp25*), 225E4 (*XAC3597/bdp11*), 228F10 (*XAC4203/bdp14*), and 295D8 (*XAC4264/bdp15*) mutants. The disrupted genes in these mutants shared no significant similarity to any motility-related genes or regulators (see above), suggesting that their impaired motility may be mainly due to their altered EPS and/or LPS production. The *gumD* mutant revealed significant reduction both in swimming and swarming motility (Supporting Information Figure S4), which is consistent with the reports that xanthan EPS is required for a type of flagellum-independent motility in *Xac* [19] and *X. campestris* pv. *campestris* [38]. Moreover, the reduced swimming phenotype in these mutants may be caused by an altered LPS. This result is consistent with our recent observations in the *wxacO* and *rfbC* mutants [15].

### Novel signaling and regulatory factors in biofilm formation

As mentioned above, we uncovered a total of seven *bdp* genes, *XAC0494/bdp18/rbfS, XAC0721*/*bdp2, XAC1499/bdp20, XAC1778/bdp21*, *XAC2018/bdp6, XAC2670/bdp22* and *XACa0007/bdp16*, associated with certain signaling and regulation pathways. Notably, both the *XAC0721*/*bdp2* and *XAC1778/bdp21* genes were involved in EPS and LPS production as well as cell motility (Table 1; Fig. 2), both the *XAC0494/bdp18/rbfS* and *XAC1499/bdp20* genes were involved in EPS production and cell motility (Table 1), and both the *XAC2018/bdp6* and *XAC2670/bdp22* genes were associated with cell motility (Table 1). The *XACa0007/bdp16* gene was involved only in EPS production (Table 1).

**Figure 3:**
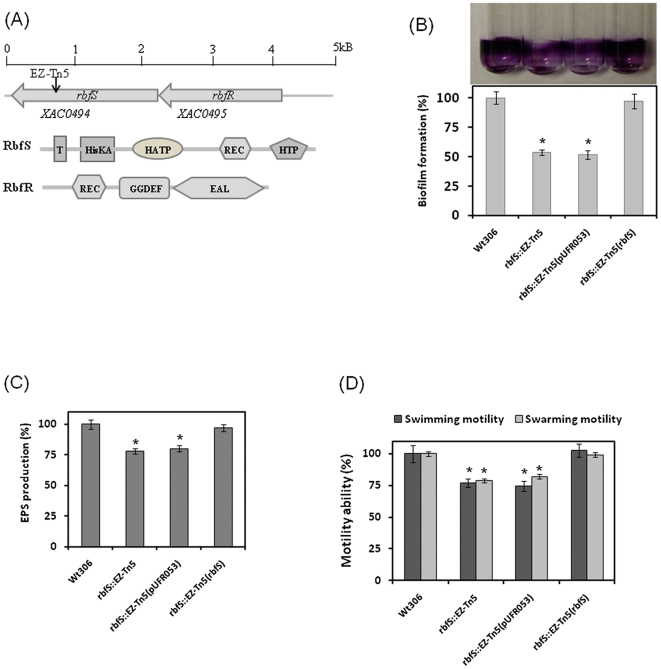
The *Xac* RbfS/RbfR two-component system plays a role in biofilm formation. (A) Genetic organization and domain structures of the RbfS sensor kinase and the RbfR response regulator. Gene orientation is indicated by arrows. Domain structure prediction was conducted using the SMART program (http://smart.embl-heidelberg.de). Abbreviations: T, transmembrane domain; HisKA, histidine kinase A domain; HATP, histidine kinase-like ATPase domain; REC, receiver domain; HTP, histidine phosphotransfer domain; GGDEF, GGDEF domain; EAL, EAL domain. (B) Restoration of biofilm formation by genetic complementation of the EZ-Tn5 insertion in rbfS. Quantification measurements of the glass tube biofilm assay are shown graphically. (C) Measurement of extracellular polysaccharide (EPS) production by strain 306 and its derivatives. (D) Cell motility test of strain 306 and its derivatives. Wt306: wild-type strain 306; rbfS::EZ-Tn5: *rbfS* mutant; rbfS::EZ-Tn5(pUFR053): *rbfS* mutant complemented with empty vector pUFR053 without the *rbfS* gene; rbfS::EZ-Tn5(rbfS): *rbfS* mutant complemented with the wild-type *rbfS* gene. All experiments were repeated three times with at least three replicates. Averages and standard errors from one of three representative experiments with similar results are shown. Wild-type strain 306 was set as equal to 100%. Significance was tested by Student's *t-*test (* indicates significant difference from wild-type strain 306 at *P*<0.01).

Interestingly, *XAC0494/bdp18/rbfS* (designated as *rbfS*, for regulation of biofilm formation, sensor) encodes a two-component system sensor protein of 769 amino acids with unknown function [5]. Based on BioCyc Transcription-Units prediction [25], *XAC0495* and *rbfS* form one transcription unit (Fig. 3A), suggesting that they may be functionally related. Further analysis revealed that RbfS contains a 23 amino acid signal peptide, a transmembrane domain, a histidine kinase A domain, a histidine kinase-like ATPase domain, a cheY-homologous receiver domain, and a histidine phosphotransfer domain (Fig. 3A). *XAC0495* encodes a two-component regulator protein containing an N-terminal receiver domain, a GGDEF domain and an EAL domain at the C-terminal (Fig. 3A). These findings strongly suggest that *rbfS* and *XAC0495* constitute a two-component signaling system. Therefore, for the convenience of discussion and for consistency with *rbfS*, *XAC0495* was designated as *rbfR* (for regulation of biofilm formation, regulator). Orthologs of the RbfS/RbfR system were found in most *Xanthomonas* species in the GenBank database and are highly homologous, with >90% amino acid identity (data not shown). The functions of RbfS/RbfR homologs have not yet been determined, with the exception of one *rbfR* ortholog, *XCC0484* in *X. campestris* pv. *campestris* strain ATCC33913, which has 92% amino acid identity and has been suggested to be involved in the general stress response of this strain [6].

The *XAC0721/bdp2* gene encodes an endonuclease/exonuclease/phosphatase domain-containing protein and is a member of a family of proteins that include Mg-dependent endonucleases and a number of phosphatases putatively involved in intracellular signaling [39]. The product of *XAC1778/bdp21* is a MASE1 domain-containing sensor kinase. MASE1-containing sensor kinases were found in various bacteria, including *E. coli* [40]. Interestingly, the homologous UhpB in *E. coli* was characterized to be involved in regulating the expression of the UhpT glucose-6-phosphate transporter, and it coordinately functions in sugar transportation [40]. No XAC1778 homolog in *Xanthomonas* spp. has been characterized, and this needs further research. The *XACa0007/bdp16* locus is located on the pXAC33 plasmid [5] and was annotated as a 139 amino acid hypothetical protein with similarity to protein kinase C (PKC) proteins. PKC is a ubiquitous phospholipid-dependent serine/threonine kinase that plays a key role in signal transduction and is involved in the regulation of numerous cellular processes, including a wide variety of biological responses to stimuli [41]. However, its role has not been reported in EPS production or biofilm formation.

In addition, both *XAC2018/bdp6* and *XAC1499/bdp20* gene products contain a predicted XRE-like HTH DNA-binding domain (Table 1). The XRE DNA-binding protein family is a large family of transcriptional regulators that may act as activators and/or repressors in a variety of bacteria [42]. For example, *mqsA* in *E. coli* encodes an antitoxin containing an XRE HTH domain that positively regulates the transcription expression of *mqsR (b3022)*, which is a motility and quorum-sensing regulator involved in biofilm formation [43], [44]. *XAC1499/bdp20* was found to be involved in EPS production and cell motility, while *XAC2018/bdp6* is only involved in cell motility (Table 1). The putative XAC2018*/*Bdp6 and XAC1499/Bdp19 proteins exhibited only a slight similarity (12% identity in amino acid sequence), which may explain the functional differences between the two genes. The product of *XAC2670/bdp23* was previously annotated as an alginate biosynthesis protein [5]. A BLASTP analysis revealed that this protein was moderately similar (30–40% identity in amino acid sequence) to two-component system sensor proteins in other bacteria, and domain analysis suggested that it contained a signal peptide, three transmembrane domains, and a histidine kinase domain followed by a histidine kinase-like ATPase domain. This evidence strongly suggests that XAC2670/Bdp23 is a two-component system sensor protein. Interestingly, our data suggest that XAC2670/Bdp23 is involved in the regulation of cell motility but not the production of EPS (Table 1). In the *Xac* strain 306 genome [5], no adjunct cognate response regulator of XAC2670 occurred. The adjacent genes upstream from *XAC2670* make up a pilus gene cluster encoding type IV pilus assembly proteins [5]. Taken together, these findings suggest that XAC2670 might play a role in the regulation of expression of genes responsible for type IV pilus biosynthesis and are thus involved in cell motility in *Xac* strain 306.

### Genetic complementation confirms the role of *XAC0482/bdp1* and *rbfS* in biofilm formation

**Figure 4:**
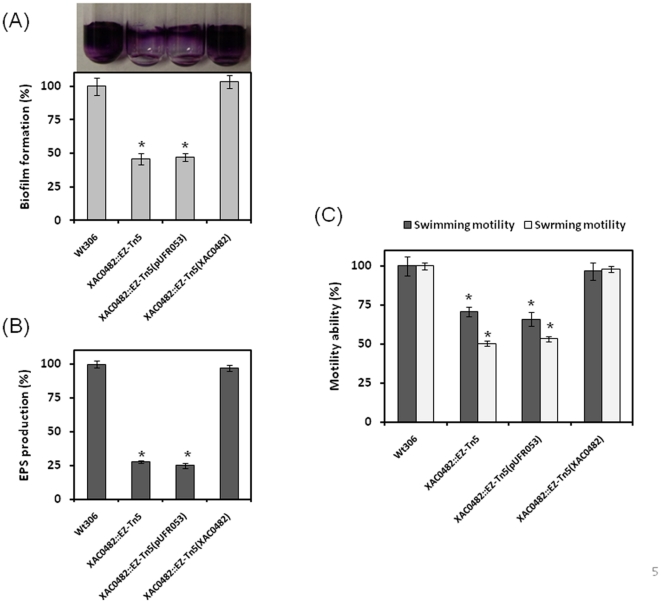
Restoration of the affected phenotypes by genetic complementation of the EZ-Tn5 insertion in *XAC0482*. (A) Quantitative measurements of *Xac* biofilm formation in wild-type strain 306 and its derivatives. (B) Measurement of EPS production by strain 306 and its derivatives. (C) Cell motility test of strain 306 and its derivatives. Wt306: wild type strain 306; XAC0482::EZ-Tn5: *XAC0482* mutant; XAC0482::EZ-Tn5(pUFR053): *XAC0482* mutant complemented with empty vector pUFR053 without the *XAC0482* gene; XAC0482::EZ-Tn5(XAC0482): *XAC0482* mutant complemented with the wild-type *XAC0482* gene. All experiments were repeated three times with at least three replicates. Averages and standard errors from one of three representative experiments with similar results are shown. Wild-type strain 306 was set as equal to 100%. Significance was tested by Student's *t-*test (* indicates significant difference from wild-type strain 306 at *P*<0.01).

To further validate our results, complementation assays were conducted for the mutants of the *XAC0482/bdp1* and *rbfS* genes. We were able to complement the *XAC0482/bdp1* and *XAC0494/rbfS* mutants with wild-type *bdp1* and *rbfS*, respectively (Fig. 3; Fig. 4). The altered phenotypes, including biofilm formation, EPS production and motility, were restored to levels comparable to those for the wild type (Fig. 3 B; C; D; Fig. 4). These findings further confirmed the linkage between the *XAC0482/bdp1* and *rbfS* genes and *Xac* biofilm formation.

## Discussion

### Isolation of *Xac* mutants defective in biofilm formation

The isolation of a large number of mutants defective in EPS production, flagellum biosynthesis, or motility validates our screening procedure for uncovering mutants because these factors have been implicated in biofilm formation on abiotic and/or biotic surfaces in other model systems [14]. This view has been further verified by the isolation of a set of 7 genes, *colR*, *fhaB*, *fliC*, *galU*, *gumD*, *wxacO* and *rbfC*, which were previously characterized to be important for *Xac* biofilm formation [10], [11], [15], [18], [19], [20]. Thus, our procedure will mostly likely validate the 33 novel biofilm-related genes identified in this study. Our approach was not complete, however, because we also missed some genes already shown to be involved in *Xac* biofilm formation, such as *rhsd*, *XAC3263*, *XAC3285* and *XAC3294* [45]. Twenty-two thousand random mutants represent a roughly fourfold coverage for a targeted insertion rate of 1 per 1,000 bp (the genome is 5.1×10^6^ bp); therefore, the screen is likely to be saturating. The variability between polystyrene and borosilicate glass and the visual inspection method used for scoring the initial screen might have precluded the capture of subtle biofilm formation deficiencies.

### Novel biofilm-related genes uncovered in this work

To the best of our knowledge, *XAC1469/bdp3, XAC1509/bdp4, XAC1923/bdp5, XAC2848/bdp7, XAC4024/bdp13* and their homologs have not previously been reported to play a role in biofilm formation in *Xanthomonas* and other bacteria. Thus, this is the first report demonstrating that these five genes are involved in biofilm formation. XAC1469/Bdp3 is a putative TatD_DNase domain-containing protein similar to members of the TatD-related DNase family (Table 1), which is a family of Mg-dependent DNases that participate in DNA replication, recombination and repair [31]. XAC1509/Bdp4 is a hypothetically cytoplasmic protein of unknown function and is only moderately conserved in *X. campestris* pv. *campestris* strain B100 and *X. campestris* pv. *vesicatoria* strain 85-10, with 80% and 40% amino acid identity, respectively. XAC1923/Bdp5 is a 334 aa hypothetical protein that contains a 41 aa signal peptide and no other conserved domains, which suggests that it may be a secreted protein. XAC1923/Bdp5 is unique to *Xac*; there are no significant homologs of XAC1923/Bdp5. The XAC2848*/*Bdp7 protein has a predicted DUF3616 domain (Pfam entry PF12275). This family of proteins is mainly observed in bacteria, and most have unknown functions. MpeA1724 in *Methylibium petroleiphilum* PM1, which shares 72% amino acid identity to XAC2848*/*Bdp7, is a lipocalin-like protein of unknown function [46]. In *E. coli*, both lipocalin-like proteins, Blc and YodA, were suggested to play a role in the adaptation of cells to certain types of stress, including cadmium stress [47], [48]. However, lipocalin-like proteins have not been reported to be involved in biofilm formation. *XAC4024/bdp13* encodes a 119 aa hypothetical protein containing a 27 aa signal peptide and a transmembrane segment at the N- terminal. This protein has a high level of similarity to outer membrane proteins with unknown functions (data not shown). Our results suggest that both XAC1469/Bdp3 and XAC1509/Bdp4 contribute to EPS production and cell motility in *Xac* and that XAC2848*/*Bdp7 contributes only to EPS production (Table 1). However, neither XAC1923/Bdp5 nor XAC4024/Bdp13 is associated with EPS production, LPS biosynthesis or cell motility in *Xac* (Table 1 and data not shown). Overall, this is the first report of a TatD-related DNase, a lipocalin-like protein, the hypothetically cytoplasmic protein XAC1509/Bdp4, the putative secreted protein XAC1923/Bdp5, and the outer membrane protein XAC4024/Bdp13 playing individual roles in biofilm formation.

### The first characterization of a HAD-like phosphatase gene in plant pathogenic bacteria

The *XAC0482/bdp1* gene encodes a hypothetical protein that possesses significant similarity to phosphatases belonging to the HAD-like hydrolase superfamily. Proteins of this family possess a variety of enzymatic activities and are widespread among the three primary kingdoms of bacteria, archaea, and eukaryotes. In *E. coli,* HAD-like phosphatases have been suggested to play important roles in carbohydrate metabolism by hydrolyzing the CO-P bond [26]. However, the vast majority of HAD-like hydrolases found in bacteria remain uncharacterized [26]. No information is available on the function of XAC0482 homologs in plant pathogenic bacteria. Our data from genetic complementation assays confirm that XAC0482 is involved in EPS production and subsequent cell motility (Table 1; Fig. 3) but not in LPS biosynthesis (Fig. 2, Lane 3). Thus, this protein may contribute to *Xac* biofilm formation through promoting the production of EPS and subsequent cell motility via unknown mechanisms. To our knowledge, this is the first analysis of a HAD-like phosphatase in plant pathogenic bacteria.

### EPS and LPS are directly involved in *Xac* biofilm formation

Seven insertions that occurred in the *gum* gene cluster (*gumCDEFJK*) required for xanthan EPS production (Supporting Information Table S1; Supporting Information Figure S3) and insertions in 13 *bdp* genes (*bdp1-4, bdp7, bdp14-16, bdp18, bdp20, bdp21, bdp24* and *bdp28)* that individually contribute to the production of EPS (Table 1) were identified in this study. In addition, an insertion was mapped in the *galU* gene, which blocked EPS and CPS biosynthesis and led to a dramatic reduction in *Xac* biofilm formation [11]. Moreover, an additional insertion was found in the *XAC0655* locus, and it affected EPS production (Supporting Information Figure S4). The *XAC0655* gene was annotated as a sugar kinase [5], and it has a predicted PfkB domain (Pfam entry PF00294) (data not shown). The predicted XAC0655 protein reveals 92% identity in amino acid sequence to the ADKXcc (XC_0690) of *X. campestris* pv. *campestris* strain 8004 [38]. Adenosine kinase (ADK), which belongs to the PfkB family of carbohydrate and nucleoside kinases, is a purine salvage enzyme that catalyzes the phosphorylation of adenosine to generate AMP [49]. It was demonstrated that a mutation in *adk*_Xcc_ affects EPS production in strain 8004 [38], which is consistent with our observation in the *XAC0655/adk_Xac_* gene mutant (Supporting Information Figure S4). These findings suggest that *XAC0655* may contribute to biofilm formation by acting as an ADK in *Xac*. Therefore, this gene was named *adk_Xac_*. Taken together, these findings are consistent with the idea that EPS is required for structured biofilm formation in a large number of bacteria [14], [16]. Given that *X. campestris* pv. *campestris* produces at least two types of EPS in rich media, including xanthan gum and others [50], and that a mutation in *gumD* abolished *Xac* production of xanthan [20], we hypothesized that *Xac* produces at least two types of EPS in rich growth media. To investigate this possibility, we examined the total EPS production of a *gumD* mutant. The result showed that the *gumD* mutant, compared with wild-type strain 306, has an 80% reduction in EPS production, but EPS was not abolished when cultured in nutrient broth (NB, Difco; Detroit, IL) supplemented with 2% glucose (Supporting Information Figure S4). This observation suggests that *Xac,* under the present experimental conditions, may produce at least two types of EPS. The major type of EPS is xanthan, which is encoded by the *gum* genes, and other types of EPS are encoded by one or more *bdp* genes. These other types of EPS might be important constituents of the biofilms formed by *Xac* because xanthan is not the sole polysaccharide implicated in biofilm formation in *X. campestris* pv. *campestris* [50].

As one of the major polysaccharide components on cell surfaces of Gram-negative bacteria, LPS has been found to be directly involved in biofilm formation in diverse bacteria, including *E. coli* O157:H7 [22] and *Pseudomonas aeruginosa* [33]. We uncovered seven *bdp* genes associated with LPS synthesis in *Xac*, including *XAC0721*/*bdp2*, *XAC1778/bdp21*, *XAC2848/bdp7*, *XAC3110/bdp24*, *XAC3364/bdp8, XAC3576/bdp9*, and *XAC4203/bdp14* (Fig. 2). We also identified nine EZ-Tn5 insertions in the putative LPS biosynthesis gene cluster in *Xac* strain 306 (*XAC3591*, *XAC3593, XAC3595*, *XAC3596/wxacO*, *XAC3597, XAC3598/rfbC, wzt, wzm* and *metB*) [5] (Supporting Information Figure S3). These findings strongly suggest that LPS is directly involved in *Xac* biofilm formation; more recently, we have more accurately described the role of LPS in structured *Xac* biofilm formation [15].

### Extracellular proteins and eDNA participate in biofilm formation

The structure and physiology of bacterial biofilms reveal a dramatic diversity of different species and environments. Typically, biofilms contain extracellular matrices consisting of polysaccharides, proteins, membrane vesicles, and extracellular DNA (eDNA) [14], [16]. Many different strains of *Xanthomonas* spp. and related genera are able to produce biofilms under certain culture conditions [10], [11], [12], [13], [51], [52], but little is known about their composition or structure except that xanthan EPS is commonly a major component of biofilm matrices and that the Xag-type EPS is implicated in biofilm formation by *X. campestris* pv. *campestris* [50]. Our most recent report demonstrated that LPS is another essential constituent of the *Xac* biofilm [15]. Interestingly, Cheng et al. [51] reported that eDNA is associated with biofilm formation in *Xylella fastidiosa*, a close relative of *Xanthomonas* spp.. Similarly, we observed a significantly decreased biofilm formation in *Xac* strain 306 after either DNase I or proteinase K treatment (Supporting Information Figure S5), implying that both eDNA and extracellular proteins are likely to be essential constituents of the *Xac* biofilm. eDNA can be released by live cells either via membrane vesicles composed of bacterial lipids or from auto-lysed cells [53]. Moreover, this work identified several genes whose products are predicted surface-associated or secreted proteins (*fhaB, vacJ* and *XAC1923*/*bdp5*), outer membrane proteins (*iroN* and *XAC4024*), or transporter components (*XAC1017* and *XAC1459*) (Supporting Information Table S1), which at least partly supports the view that both extracellular proteins and eDNA are likely to be essential constituents of *Xac* biofilms.

### Flagellum, type IV pili, bacterial chemotaxis and motility are critical for biofilm formation

Flagella and type IV pili have been implicated in biofilm formation in various bacteria by performing three potentially exclusive roles individually or collectively. They (1) enable planktonic cells to swim toward nutrients associated with a surface or toward signals generated by cells attached to a surface via flagellar-mediated chemotaxis; (2) enable bacteria to overcome repulsive forces and to initially reach a surface and allow attached, dividing bacteria to spread along a surface; and (3) function in a direct manner by physically adhering to a surface [for review, see 14]. In this work, we obtained thirteen different insertions in gene clusters required for flagellum biogenesis (*fleN, flgABFKL, flhB,* and *fliCFMQR)*, one insertion in the type IV pili biogenesis gene cluster (*pilB*), four insertions in bacterial chemotaxis-related genes (*cheA, cheY, mcpA,* and *tsr*), and two insertions in the motility-related gene, *motB* (two different copies) (Supporting Information Table S1; Supporting Information Figure S3). Moreover, the *filC* gene mutant demonstrated severely weakened motility compared to the wild-type strain 306 (Supporting Information Figure S4). Although it is not yet clear which aspect(s) of flagellar structure/function are important in biofilm development for this bacterium, these observations, along with others [19], demonstrate that flagellum- and/or pili-related motility is indeed required for *Xac* biofilm formation, which is consistent with observations in other model systems [14].

Interestingly, our results show that the 332D5 (*XAC0482/bdp1*), 411F5 (*XAC1509/bdp4*), 223G4 (*XAC3110/bdp24*), 292G4 (*XAC3364/bdp8*), 291A9 (*XAC3591/bdp25*), 225E4 (*XAC3597/bdp11*), 228F10 (*XAC4203/bdp14*), and 295D8 (*XAC4264/bdp15*) mutants were not only reduced in motility but were also impaired in EPS and/or LPS production (Table 1). No homologs of these eight genes share significant similarity to any motility-related gene or regulator (Table 1), suggesting that the impaired motility in these mutants may result mainly from their altered EPS and/or LPS production. Indeed, the *gumD* mutant revealed significantly decreased swimming and swarming motility (Supporting Information Figure S4), which is consistent with the idea that xanthan EPS is required for a flagellum-independent type of motility in *Xac* [19] and *X. campestris* pv. *campestris* [38]. Moreover, we recently observed that altered LPS leads to a reduced swimming phenotype in the *wxacO* or *rfbC* gene mutants [15]. However, further characterization is necessary for examining whether this flagellum-independent type of motility contributes to *Xac* biofilm formation and the exact role that it plays in biofilm formation.

### Plasmid genes involved in biofilm formation

In our mutational analyses, we identified three independent insertions in the *XACa0007/bdp16, XACb0001/bdp17* and *XACb0050/bdp28* genes that are located on the pXAC33 (*XACa0007/bdp16*) and pXAC64 (*XACb0001/bdp17* and *XACb0050/bdp28*) plasmids. The product of the *XACa0007/bdp16* gene is a hypothetical protein with similarity to PKC proteins. PKC is a widespread phospholipid-dependent serine/threonine kinase that plays an important role in signal transduction and is involved in the regulation of numerous cellular processes, such as cellular growth, migration and proliferation, and a wide variety of biological responses to stimuli [41]. In the human bacterial pathogen, *E. coli*, the PKC-related signal transduction pathway plays a significant role in invasion [41] and is required for bacterial adherence to host cells [54]. Results from this work suggest that *XACa0007/bdp16* is associated with the regulation of EPS production (Table 1). The *XACb0001/bdp17* gene was annotated as a 68 aa hypothetical protein that shares similarity to Radical *S*-adenosylmethionine (SAM) domain-containing proteins (data not shown). Radical SAM proteins catalyze a diversity of reactions, including unusual methylation, isomerization, sulfur insertion, ring formation, anaerobic oxidation, and protein radical formation. Additionally, these proteins function as DNA precursors in vitamin, cofactor, antibiotic and herbicide biosynthesis as well as in biodegradation pathways [55]. Our data reveal that XACb0001/Bdp17 is not associated with EPS production or cell motility; thus, the role of XACb0001/Bdp17 in biofilm formation remains unknown. The *XACb0050/bdp28* locus encodes an ISxac2 transposase [5]. Previous investigations have demonstrated that bacterial transposases are involved in biofilm formation. The IS256 transposase in *Staphylococcus epidermidis* influences biofilm formation, either by insertion into regulatory genes or by modulating biofilm gene expression [32]. The results from our study suggest that *XACb0050/bdp28* is involved in EPS production (Table 1) and thus contributes to biofilm formation. Both pXAC33 and pXAC64 plasmids are known to be critical for the virulence of *Xac* because they encode the PthA type III effector protein, which is a required pathogenicity determinant in *Xac* [1]. The results from the present study indicate that the pXAC33 and pXAC64 plasmids are associated with biofilm formation, suggesting the importance of these plasmids for the adaptation of *Xac* to adverse environmental conditions.

### Multiple signaling and regulation pathways participate in biofilm formation

As indicated above, a total of seven *bdp* genes, *XAC0494/bdp18/rbfS, XAC0721*/*bdp2, XAC1499/bdp20, XAC1778/bdp21*, *XAC2018/bdp6, XAC2670/bdp22* and *XACa0007/bdp16*, were found to be associated with signaling and regulation pathways. In addition to the seven *bdp* genes, five previously known signaling factors or regulators, including the quorum-sensing RpfC/RpfG system components of RpfC and RpfF, the ColS/ColR two-component system regulator ColR, the global regulator Clp (XAC0483), and the transcriptional activator sigma-54 factor RpoN *(*XAC1969*)*, were found to be involved in biofilm formation (Supporting Information Table S1). These data show that multiple signaling and regulation pathways are involved in biofilm formation, and these pathways can be induced by various specific extracellular or intracellular signals.

Global regulatory systems, including TCSTSs, have been reported to regulate biofilm formation in a wide variety of bacteria [14], [16]. In addition to the RpfC/RpfG, RbfS/RbfR, and ColS/ColR systems, our work identified an insertion in the *XAC1994* locus, which is a homolog of RavS. RavS is the sensor in the RavS/RavR two-component system in *X. campestris* pv. *campestris* strain ATCC33913 [56]. The product of *XAC1994* was annotated as a HrpX-related protein [5]. Further analyses revealed that XAC1994 contains one transmembrane domain, two PAS domains, one histidine kinase domain, and one histidine kinase-like ATPase domain. Its immediate downstream locus, *XAC1992,* encodes a cyclic di-GMP (c-di-GMP) phosphodiesterase A that has an N-terminal GGDEF domain, an EAL domain and a receiver domain at the C-terminal (Supporting Information Figure S6). Moreover, the XAC1994 and XAC1992 proteins revealed 90% and 99% identity in amino acid sequences to RavS (Xcc1960) and RavR (Xcc1958) of *X. campestris* pv. *campestris* strain ATCC33913, respectively. The RavS/RavR system positively regulates the production of virulence factors, including EPS, through the Clp global regulator in *X. campestris* pv. *campestris* strain ATCC33913 in response to limited levels of oxygen [56]. Notably, our study also identified a mutant with an insertion in the *clp* (*XAC0483*) locus (Supporting Information Table S1), and phenotype assays showed that both the *XAC1994* and *clp* mutants demonstrated reduced EPS production and cell motility (Supporting Information Figure S4). These findings suggest that XAC1994 and XAC1992 may constitute another two-component system in *Xac* that regulates biofilm formation through the regulation of EPS production and cell motility via Clp, similar to the process found in RavS/RavR, in *X. campestris* pv. *campestris.* Thus, *XAC1994* and *XAC1992* were renamed as *ravS_Xac_* and *ravR_Xac_,* respectively. In addition, the *XAC2670/bdp23* gene encodes a two-component system sensor protein that plays a role in the regulation of cell motility.

We also uncovered five individual signaling factors or regulators that are involved in biofilm formation. The XAC0721/Bdp2 protein is a member of a family of proteins, including Mg-dependent endonucleases and a number of phosphatases putatively involved in intracellular signaling [39]. XAC1778/Bdp21 is a MASE1 domain-containing sensor kinase involved in sugar transportation. XAC2018/Bdp6 and XAC1499/Bdp20 have XRE DNA-binding domains containing transcriptional regulators that are involved in cell motility and EPS production (Table 1), and XACa0007/Bdp16, which is a PKC protein, plays a role in the signal transduction pathway(s) related to EPS production.

**Figure 5:**
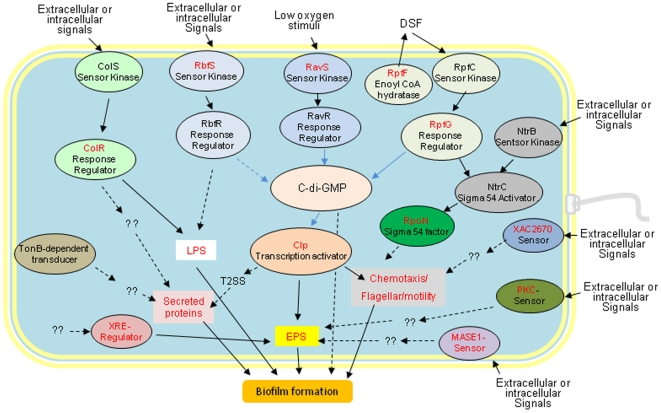
A schematic illustration of the global regulation in biofilm formation by *X. axonopodis* pv. *citri (Xac)*. The principle pathways are presented, including experimentally determined systems and other members that were not discussed in detail here. The global regulator in Xac is the cyclic di-GMP second messenger. Cyclic di-GMP is degraded by the HD-GYP or EAL domain-containing proteins via RpfC/RpfG, RavS/RavR, and RbfS/RbfR TCSTSs; by cell-to-cell communication; and/or other methods. Cyclic di-GMP levels affect the activity of the Clp transcriptional activator and subsequently affect EPS, cell motility, extracellular proteins/enzymes, and final biofilm formation. Genes identified in this study are presented in red, while genes that were previously reported in xanthomonads are presented in black. Black arrows indicate positive regulation, and blue arrows indicate negative regulation. A dotted line suggests a hypothesized connection that needs to be probed by further experimentation.

Taken together, our results significantly advance our understanding of the regulatory network of biofilm formation in the citrus pathogen, *Xac* (Fig. 5). This network contains at least four different regulatory systems that are essential for the regulation of biofilm formation. In the diffusible signal factor (DSF)-dependent RpfC/RpfG signaling system, the RpfC/RpfG proteins may play a role similar to their homologs in *X. campestris* pv. *campestris* [52], [57], [58], [59]. In *Xac,* the RpfC sensor kinase interacts with RpfF to control DSF signal generation [60], and upon reaching a threshold concentration (a tightly controlled or balanced amount) in the extracellular environment, a DSF signal is transferred through RpfC to the RpfG response regulator by a conserved phosphorelay mechanism [60]. Activated RpfG modulates the physiological concentrations of the c-di-GMP second messenger via the HD-GYP domain and its interactions with some GGDEF proteins [60]. Relatively higher levels of c-di-GMP promote biofilm formation and inhibit DNA binding by the Clp transcriptional activator [61], but decreases in levels of c-di-GMP promote motility [62], [63], [64] and activate Clp, which directly or indirectly regulates the expression of genes involved in EPS production, extracellular enzyme synthesis, and protein metabolism [58]. These gene products are suggested to be required for mature biofilm formation. Interestingly, it was observed elsewhere that both DSF overproduction and non-production adversely affect the formation of a structured biofilm in *X. campestris* pv. *campestris* [52]. One possible reason for this adverse effect is that RpfG negatively regulates the glycosyltransferase system involved in synthesizing polysaccharide adhesins that are essential for biofilm formation [50]. Additionally, RpfG may control another set of genes implicated in biofilm dispersal [57], [65]. Moreover, RpfG may interact with NtrC, a response regulator in the NtrB/NtrC two-component system, and, in this way, contribute to the NtrC-mediated regulation of sigma 54 factor-dependent promoters [60] and subsequent regulation of genes involved in biofilm formation. Another signaling pathway is the RavS_Xac_/RavR_Xac_ two-component regulatory system. The RavS/RavR-mediated low-oxygen tension-sensing pathway in *X. campestris* pv. *campestris* positively regulates the production of virulence factors, including EPS, through modulation of the intracellular levels of c-di-GMP and Clp in a manner similar to the DSF-dependent RpfC/RpfG signaling system [56]. Similarly, a limited level of oxygen in the environment, such as in a growing biofilm microcolony, may activate the RavS/RavR regulatory pathway to promote EPS production and thus help biofilm maturation in *Xac*. A third signaling pathway is the novel RbfS/RbfR regulation pathway. In this system, the RbfS sensor detects certain extracellular or intracellular signals and transfers them to the RbfR response regulator. The RbfR protein contains N-terminal REC and GGDEF domains and a C-terminal EAL domain (Fig. 4A). The activated GGDEF and/or EAL domains interact with c-di-GMP, modulating the intracellular levels of c-di-GMP and Clp and subsequently affecting the expression of genes related to biofilm formation. Additionally, the ColS/ColR two-component system regulates biofilm formation. Our recent work indicated that the ColS/ColR functions in biofilm formation by modulating LPS biosynthesis in response to various environmental stimuli [18]. Taken together, the data suggest cumulative regulation mechanisms whereby *Xac* may coordinate RpfC/RpfG-dependent quorum-sensing signaling, RavS/RavR-mediated low-oxygen tension sensing and the novel RbfS/RbfR system to regulate biofilm development through modulating intracellular levels of the Clp global regulator and the c-di-GMP second messenger. In addition, the data suggest that there are individual signaling factors or regulators that take part in some unknown signaling pathways to regulate biofilm formation in response to various specific extracellular or intracellular stimuli.

### A model for biofilm formation in *Xanthomonas*

**Figure 6:**
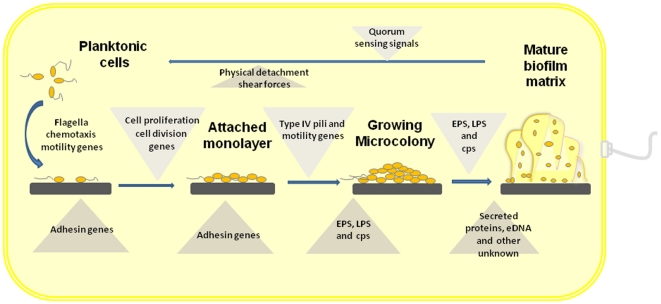
Schematic simulation of the multistage biofilm formation process in *X. axonopodis* pv. *citri*. The counterclockwise cycle initiates with the attachment of individual cells developing into a three-dimensionally complex, multicellular biofilm matrix. Eventual dispersal releases a subpopulation of cells from the biofilm matrix into the planktonic phase.

This study also dramatically enhances our understanding of the genetic process of *Xanthomonas* biofilm formation, as described in Fig. 6. In this model (Fig. 6), bacteria respond to certain extracellular or intracellular signals, such as the quorum-sensing DSF signals and/or nutrient elements, by using flagellum-mediated chemotaxis and motility to move toward, reach and attach to appropriate surfaces with the help of adhesin proteins, such as FhaB. The presence of the FhaB adhesin promotes a stable adherence to the surface. With access to sufficient levels of nutrients and oxygen, genes such as *minD, mrdB* and *nrdF* (Supporting Information Table S1), which encode proteins involved in DNA replication, modification, processing and degradation and are linked to adaptation and differentiation, may be activated, and cells can begin to divide on the surface. Additionally, through cell proliferation and division, bacteria spread along the surface through flagellum- and/or type IV pili-mediated chemotaxis/motility, and a monolayer consisting of cells attached to each other emerges and attaches firmly to the surface through the generation of more adhesin proteins. As cells continue to grow on top of one another, a microcolony is formed, and the metabolism of cells near the surface may restrict the diffusion of oxygen and nutrients to the cells at the bottom of the microcolony. Some of these cells may adapt to these changes in local environment, and this leads to phenotypic heterogeneity within this microcolony. The relatively low levels of oxygen and nutrients activate the response regulators of two-component regulatory systems, such as RavS_Xac_/RavR_Xac_, RbfS/RbfR and ColS/ColR, and consequently, a proportion of cells are activated to express higher levels of EPS, including both xanthan gum and the unknowns. Additionally, LPS, secreted and/or extracellular proteins, and eDNA are all secreted from the cell. These compounds help cells attach to each other and promote the vertical growth of the microcolony. The increased vertical topology may improve access to oxygen and nutrients, which in turn promotes the topological growth of the microcolony and thus the establishment of macrocolonies and a mature biofilm matrix. Indeed, our recent observation of confocal scanning laser micrographs of *Xac* strain 306 examined the maturation of this bacterium from early-attached cells to the formation of a mature biofilm [15]. Bacterial cells in the biofilm matrix adapt to this new local environment, creating even further heterogeneity within the biofilm. Finally, a proportion of cells or fragments of a microcolony detach and disperse from the biofilm matrix in response to a programmed set of events directed by the DSF signals in a cell-to-cell manner [57, 65, Y Guo and N Wang, unpublished data in this lab) or as a result of physical shear forces.

### Conclusions

In conclusion, we report a genome-wide identification of genes related to *Xac* biofilm formation. We uncovered 92 biofilm-related genes, of which 17 are previously unknown, novel genes and 16 have only putative functions. The putative products of 5 novel genes, including a TatD-related DNase, a lipocalin-like protein, a hypothetically cytoplasmic protein XAC1509/Bdp4, a secreted protein XAC1923/Bdp5, and an outer-membrane protein XAC4024/Bdp13, were described for the first time as being involved in biofilm formation. Furthermore, two of these 92 biofilm-related genes, *XAC0482/bdp1* and *rbfS*, were experimentally confirmed to contribute to biofilm formation. Our findings indicate that EPS, LPS and flagellum-dependent and flagellum-independent motility are required for the formation of a mature biofilm. Additionally, extracellular proteins and eDNA also contribute to biofilm formation. Based on our data, and in conjunction with others, a comprehensive developmental process and a global regulatory network of *Xac* biofilm formation has been postulated (Figs. 5 & 6). This work is the first report on a genome-wide scale of the genetic processes of biofilm formation in a plant pathogenic bacterium, and it provides new insight into the genetic determinants and regulation mechanisms of biofilm formation. Further analyses of these biofilm formation-related genes and the pathways involved will provide a better understanding of the process of biofilm formation and adaptation in *Xac*. This, in turn, should aid in the development of more effective control strategies for citrus canker.

## Materials and Methods

### Bacterial strains, growth conditions and plasmids

The key strains and plasmids used in this work are listed in Supporting Information Table S2. The *E. coli* strains were cultured at 37°C in Luria-Bertani (LB) medium. Wild-type *Xac* strain 306 (rifamycin resistant) [66] and mutant strains were grown at 28°C in nutrient broth (NB, Difco; Detroit, IL) or on nutrient agar (NA, Difco; Detroit, IL). When necessary, antibiotics were added at the following concentrations in growth media: ampicillin (Ap), 50 µg/ml; chloramphenicol (Cm), 35 µg/ml; gentamicin (Gm), 5 µg/ml; kanamycin (Km), 50 µg/ml; or rifamycin (Rif), 50 µg/ml.

### Mutant generation and screening for biofilm-defective phenotype

A transposon mutant library of *Xac* strain 306, which contains 22,000 clones, was previously constructed using the EZ-Tn5™ <R6Kγori/KAN-2>Tnp Transposome™ Kit (Epicentre, Madison, WI) [11]. For rapid initial screening, a polystyrene 96-well plate assay was performed as described previously [15]. Candidate bdp mutants that passed the initial screening were subsequently subjected to a stringent screening using a quantitative biofilm assay in borosilicate glass tubes as described by Guo et al. [11]. The test was repeated three times independently. Mutants that revealed biofilm-defective phenotypes were selected for further characterization.

### EZ-Tn5 transposon copy number determination

Genomic DNA was extracted from strains of *Xac* using a Wizard genomic DNA purification kit according to the manufacturer's protocols (Promega, Madison, WI), digested with *Sac*I (which has no target site in the EZ-Tn5 transposon), subjected to electrophoresis on a 0.8% agarose gel, and transferred to a positively charged nylon membrane (Roche, Indianapolis, IN) using standard procedures [67]. A DNA fragment of the kanamycin-resistance gene of the EZ-Tn5 transposon was amplified with primers Kan-1 and Kan-2 (Supporting Information Table S3) and used as the probe. Probe labeling, hybridization, and immunological detection were performed using a digoxigenin (DIG)-High Prime II DNA labeling and detection starter kit following the manufacturer's instructions (Roche, Indianapolis, IN).

### Identification of the EZ-Tn5 flanking sequence

The genomic sequences flanking the EZ-Tn5 transposon in bdp mutants were analyzed using the random amplification of transposon ends (RATE) PCR method [23]. Briefly, mutant genomic DNA was used as a template in a three-step PCR reaction with the Inv-1 or Inv-2 primer (Supporting Information Table S3). The first 30 cycles of the PCR reaction were performed at 55°C with a 30-second extension. The second 30 cycles were conducted at 30°C with a 30-second extension. The last 30 cycles of the amplification were conducted at 55°C with a 2-minute extension. The RATE-PCR products were then sequenced using the forward (KAN-2 FP-1) or reverse (KAN-2 RP-1) primers (Supporting Information Table S3) supplied with the EZ-Tn5™ <R6Kγori/KAN-2> Insertion Kit and analyzed using BLASTN against the *Xac* strain 306 genome sequence [5].

### Construction of plasmids for genetic complementation

Plasmids used for genetic complementation were constructed as follows. The corresponding primers (Supporting Information Table S3) were designed to incorporate upstream *Bam*HI and downstream *Hin*dIII restriction sites flanking the coding sequence and were used to amplify *XAC0482* (*bdp1*) and *XAC0494* (*rbfS*) from *Xac* wild-type strain 306 genomic DNA. The PCR product was cloned into a pGEM®-T Easy vector following the manufacturer's instructions (Promega, Madison, WI). Following this, the *Bam*HI-*Hin*dIII fragment was isolated and ligated into the pUFR053 complementary vector [68] to construct the pUF-0482 and pUF-rbfS plasmids (Supporting Information Table S2) for genetic complementation. Plasmids were introduced into the appropriate biofilm mutants by triparental mating as described previously [15].

### EPS quantitative determination

To estimate total EPS production, bacterial strains were cultured in 50 ml of NB liquid medium containing 2% (wt/vol) glucose at 28°C with shaking at 200 rpm for 24 hours. EPS was precipitated from the culture supernatant with ethanol, dried, and weighed, as described previously [50]. The experiment was repeated three times with three replicates.

### Cell motility assay

To test cell motility, 2 µL of overnight cultures in NB (OD600 of 1.5) of each *Xac* strain was spotted onto NA plates containing 0.3% (wt/vol) agar (Difco, Franklin Lakes, NJ) for the swimming motility assay or 0.7% (wt/vol) agar for the swarming motility assay. Plates were incubated at room temperature (22–23°C) for 7 days. The diameters of the areas occupied by the strains were measured, and the values were used to indicate the motility of *Xac* strains. The experiment was repeated three times with three replicates.

### LPS analysis

*Xac* strains were cultured overnight at 28°C in NB liquid medium with shaking at 200 rpm. Five mL of cultures at the exponential stage was collected, and the LPS samples were extracted as previously described [15]. LPS was separated using sodium dodecyl sulfate polyacrylamide gel electrophoresis (SDS-PAGE) and visualized using silver staining following the manufacturer's instructions (Bio-Rad Laboratories, Inc., Hercules, CA). Standard LPS from *Salmonella enterica* serovar Typhimurium was obtained from Sigma. The test was independently performed three times.

## Supporting Information

Figure S1 Southern hybridization analysis of genomic DNA extracted from biofilm-defective mutant strains of *Xanthomonas axonopodis* pv. *citri (Xac).* M: size marker, 1 Kb plus (Promega); Lane 1: wild-type strain 306; Lanes 2–18: mutant strains; Lane 12: double-transposon insertion mutant. The genomic DNA of *Xac* strains was digested with *Sac*I, blotted onto a nylon membrane and hybridized with a kanamycin-resistant gene as the probe.
(TIF)

Figure S2 Distribution of biofilm-formation-related genes identified in this work in the chromosome genome and plasmids of *Xanthomonas axonopodis* pv. *citri* strain 306.
(TIF)

Figure S3 Genetic organization of biofilm-formation-related gene clusters in *Xanthomonas axonopodis* pv. *citri* strain 306. (a) Gum genes cluster, (b) LPS biosynthesis genes cluster, (c) EPS and LPS precursors biosynthesis gene cluster, (d) hemagglutinin coding genes cluster, (e) Chemotaxis/flagellum/motility genes cluster, and (f) pili gene cluster. Black triangles indicate insertion sites of the EZ-Tn5 transposon.
(PDF)

Figure S4 Assays for EPS production (A) and cell motility (B) of biofilm-defective mutants of *Xanthomonas axonopodis* pv. *citri* strain 306. For the EPS assay, a modified ethanol deposit method was applied. For the motility test, bacterial strains were inoculated at a central point on NA plates (0.3% agar for swimming assays and 0.7% agar for swarming assays) and incubated at room temperature (approximately 23°C) for 7 days, after which colony diameters were measured. All experiments were repeated three times with three replicates. Averages and standard errors from one of three representative experiments with similar results are presented. Wild-type strain 306 was set as equal to 100%. Significance was tested by Student's *t-*test (* indicates significant difference from wild-type strain 306 at *P*<0.01). Wt306: wild type strain 306; 294E2(XAC0144/iroN): EZ-Tn5 insertion in *XAC0144*; 332D5(XAC0482/bdp1): EZ-Tn5 insertion in *XAC0482;* 283C5(XAC0483/clp); EZ-Tn5 insertion in *XAC0483;* 301B4(XAC0494/bdp18/rbfS): EZ-Tn5 insertion in *XAC0494*; 421F4(XAC0655/adk): EZ-Tn5 insertion in *XAC0655*; 285E8(XAC0721/bdp2): EZ-Tn5 insertion in *XAC0721*; 294E4(XAC0929/bdp19): EZ-Tn5 insertion in *XAC0929*; 270H4(XAC1469/bdp3): EZ-Tn5 insertion in *XAC1469*; 319B1(XAC1499/bdp20): EZ-Tn5 insertion in *XAC1499*; 411F5(XAC1509/bdp4): EZ-Tn5 insertion in *XAC1509*; 296G1 (XAC1778/bdp21): EZ-Tn5 insertion in *XAC1778*; 288D11(XAC1923/bdp5): EZ-Tn5 insertion in *XAC1923*; 212C9(XAC1994/ravS):EZ-Tn5 insertion in *XAC1994*; 290E7(XAC2018/bdp6): EZ-Tn5 insertion in *XAC2018*; 295H3(XAC2848/bdp7): EZ-Tn5 insertion in *XAC2848*; 291G5(XAC2670/bdp23): EZ-Tn5 insertion in *XAC2670*; 223G4(XAC3110/bdp24): EZ-Tn5 insertion in *XAC3110*; 292G4(XAC3364/bdp8): EZ-Tn5 insertion in *XAC3364*; 207A6(XAC3576/bdp9): EZ-Tn5 insertion in *XAC3576*; 291A9(XAC3591/bdp25): EZ-Tn5 insertion in *XAC3591*; 225E4(XAC3597/bdp11): EZ-Tn5 insertion in *XAC3597*; 409D8(XAC4024/bdp13): EZ-Tn5 insertion in *XAC4024*; 228F10(XAC4203/bdp14): EZ-Tn5 insertion in *XAC4203*; 302B11(XAC4249/xynA): EZ-Tn5 insertion in *XAC4249*; 295D8 (XAC4264/bdp15): EZ-Tn5 insertion in *XAC4264*; 347D10(XAC4344/vacJ): EZ-Tn5 insertion in *XAC4344*; 288C6(XACa0007/bdp16): EZ-Tn5 insertion in *XACa0007*; 423H3(XACb0001/bdp17): EZ-Tn5 insertion in *XACb0001;* 419F10(XACb0050/bdp28): EZ-Tn5 insertion in *XACb0050;* 276B8(XAC1975/fliC): EZ-Tn5 insertion in *XAC1975/fliC,* and 257H6(XAC2583/gumD): EZ-Tn5 insertion in *XAC2583/gumD.*
(PDF)

Figure S5 DNase I and Proteinase K effect on bifilm formation by *Xanthomonas axonopodis* pv. *citri* strain 306. Biofilm assay showing the repartition of the strains with different treatment. W, Wild-type strain 306; W+D, Wild-type strain 306 with DNase I (20 µg/mL Sigma, St. Louis, MO); W+P, Wild-type strain 306 with Proteinase K (10 ug/ml; Sigma); CK-, NB medium without bacteria inoculation. The experiment was repeated three times with eight replicates each time. Averages and standard errors from one representative experiment of three with similar results are presented. Wild-type strain 306 was set equal to 100%. Significance was tested by Student's *t* test (* indicates significant difference from wild-type strain 306 at *P*<0.01). The inset shows a typical polystyrene microtiter dish assay plate stained with crystal violet.
(TIF)

Figure S6 Genetic organization and domain structures of the RavS_xac_/RavR_xac_ system in *Xanthomonas axonopodis* pv. *citri* strain 306. RavS_xac_ is a sensor kinase and RavR_xac_ is the response regulator. Gene orientation is indicated by arrow. Domain structure prediction was done using the SMART program (http://smart.embl-heidelberg.de). Domain Symbol: T, transmembrane domain; PAS, PAS domain; HKA, histidine kinase A domain; HATP, histidine kinase-like ATPase domain; GGDEF, GGDEF domain; EAL, EAL domain; REC, receiver domain.
(TIF)

Table S1 Biofilm related genes identified from *Xanthomonas axonopodis* pv. *citri* strain 306 in this study.
(DOC)

Table S2 Bacterial strains and plasmids used in this study^a^.
(DOC)

Table S3 Primers used in this study.
(DOC)
