# Supplementary figures and images for: Genome-Wide Mutagenesis of Xanthomonas axonopodis pv. citri Reveals Novel Genetic Determinants and Regulation Mechanisms of Biofilm Formation
Source: PLoS One. 2011 Jul 5;6(7):e21804. doi: 10.1371/journal.pone.0021804 (PMC3130047; doi:10.1371/journal.pone.0021804)

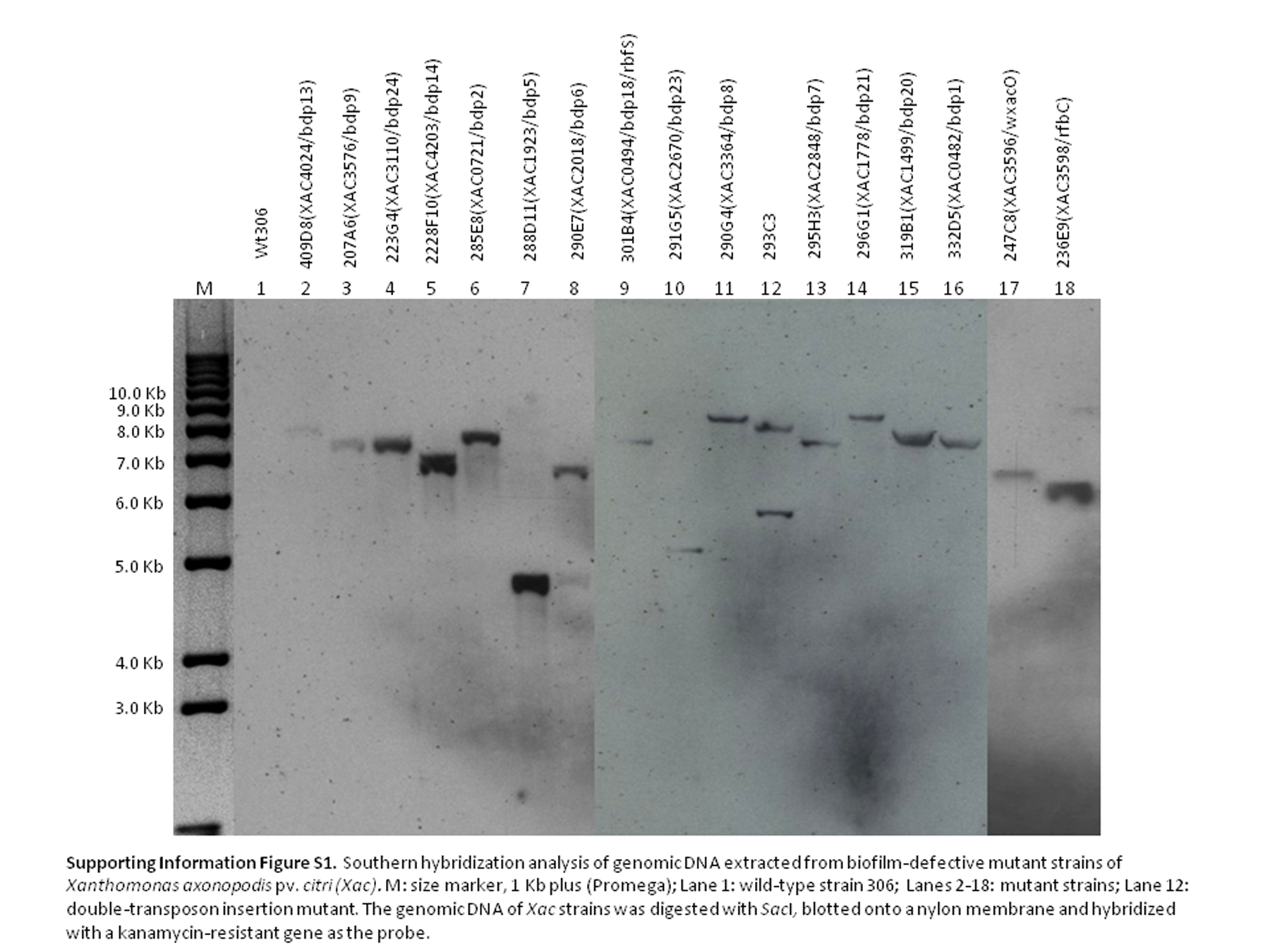

Supplement: Figure S1 — Southern hybridization analysis of genomic DNA extracted from biofilm-defective mutant strains of Xanthomonas axonopodis pv. citri (Xac). M: size marker, 1 Kb plus (Promega); Lane 1: wild-type strain 306; Lanes 2–18: mutant strains; Lane 12: double-transposon insertion mutant. The genomic DNA of Xac strains was digested with SacI, blotted onto a nylon membrane and hybridized with a kanamycin-resistant gene as the probe. (TIF) [file pone.0021804.s001.tif]

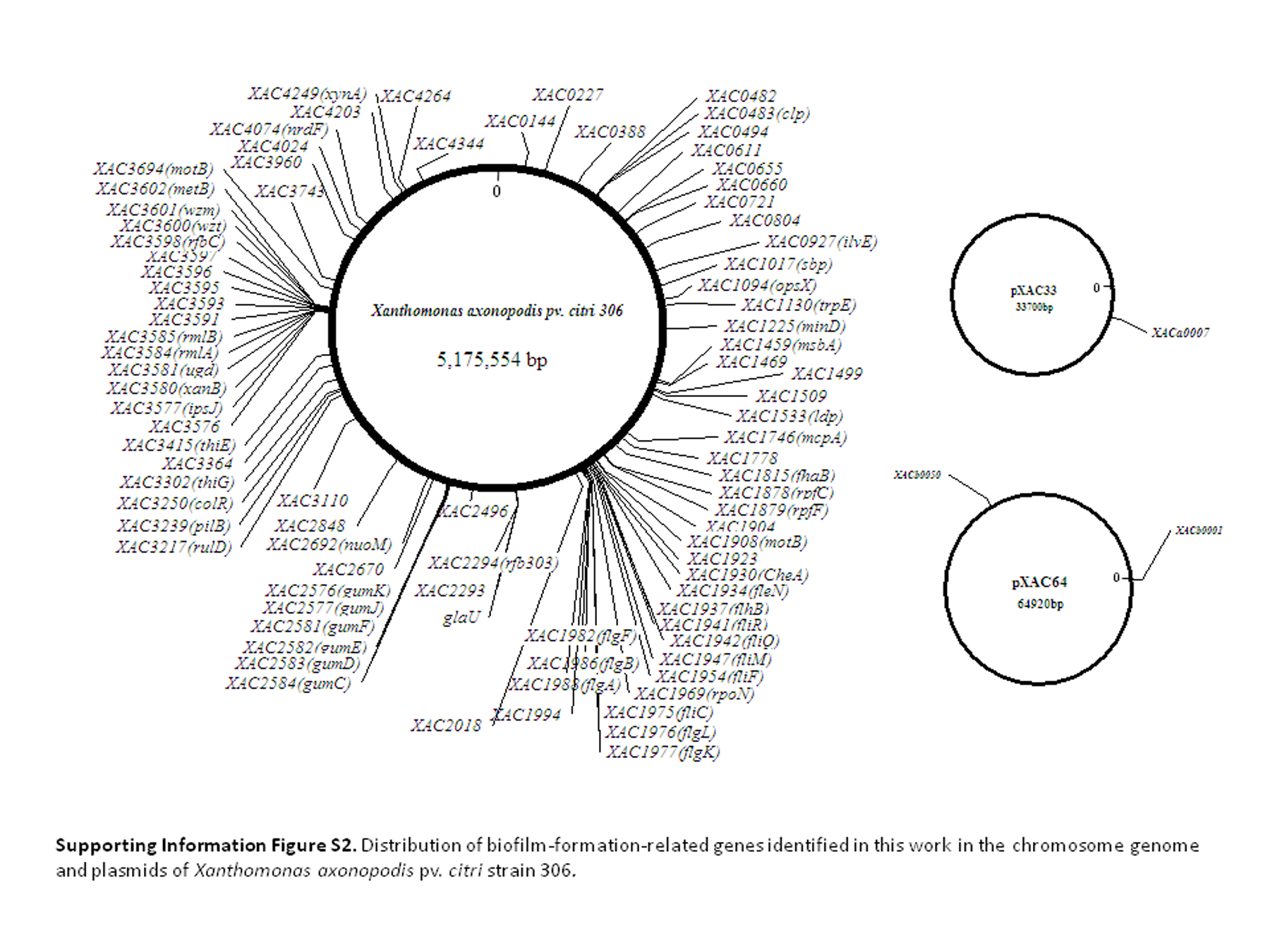

Supplement: Figure S2 — Distribution of biofilm-formation-related genes identified in this work in the chromosome genome and plasmids of Xanthomonas axonopodis pv. citri strain 306. (TIF) [file pone.0021804.s002.tif]

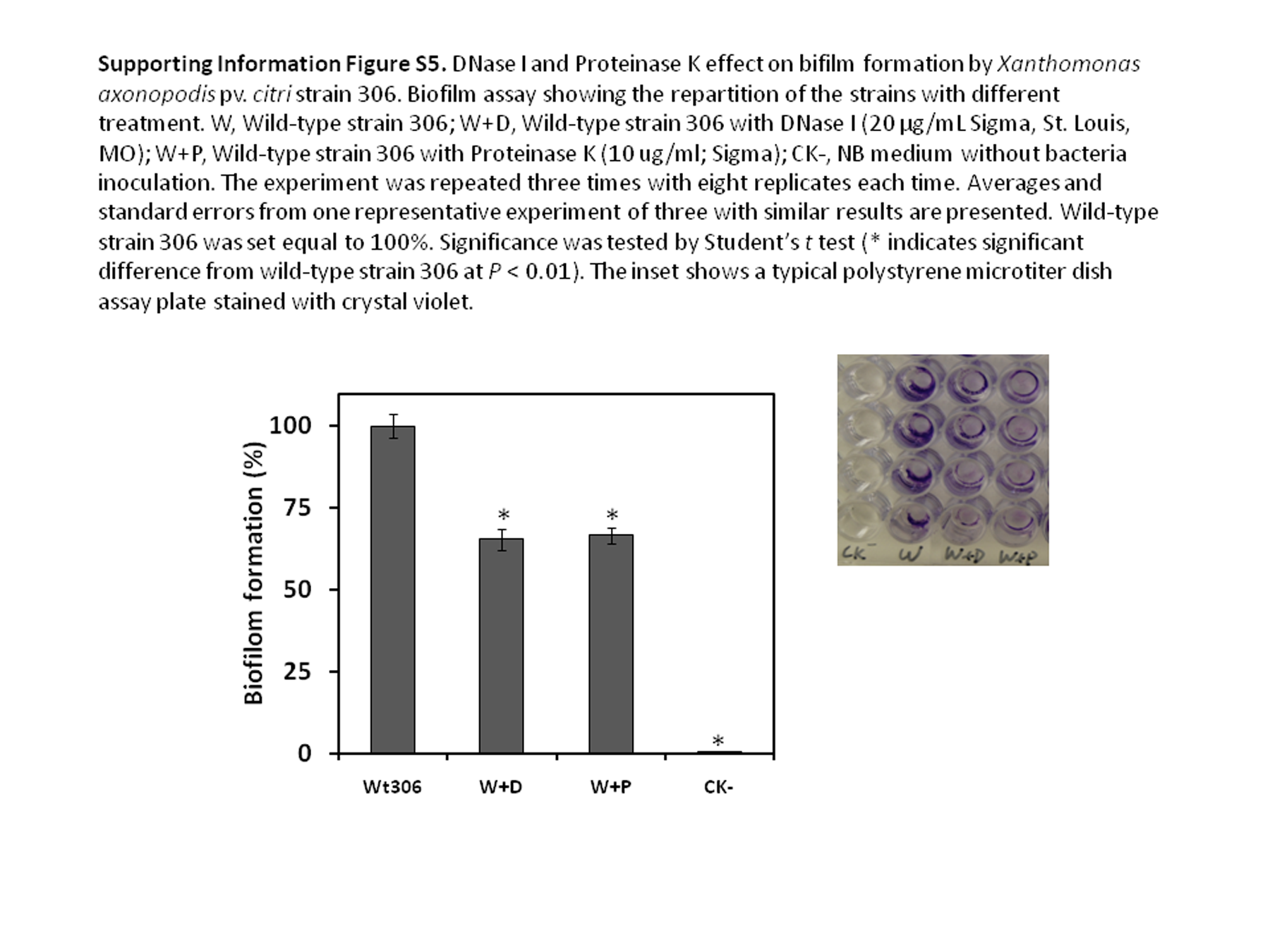

Supplement: Figure S5 — DNase I and Proteinase K effect on bifilm formation by Xanthomonas axonopodis pv. citri strain 306. Biofilm assay showing the repartition of the strains with different treatment. W, Wild-type strain 306; W+D, Wild-type strain 306 with DNase I (20 µg/mL Sigma, St. Louis, MO); W+P, Wild-type strain 306 with Proteinase K (10 ug/ml; Sigma); CK-, NB medium without bacteria inoculation. The experiment was repeated three times with eight replicates each time. Averages and standard errors from one representative experiment of three with similar results are presented. Wild-type strain 306 was set equal to 100%. Significance was tested by Student's t test (* indicates significant difference from wild-type strain 306 at P<0.01). The inset shows a typical polystyrene microtiter dish assay plate stained with crystal violet. (TIF) [file pone.0021804.s005.tif]

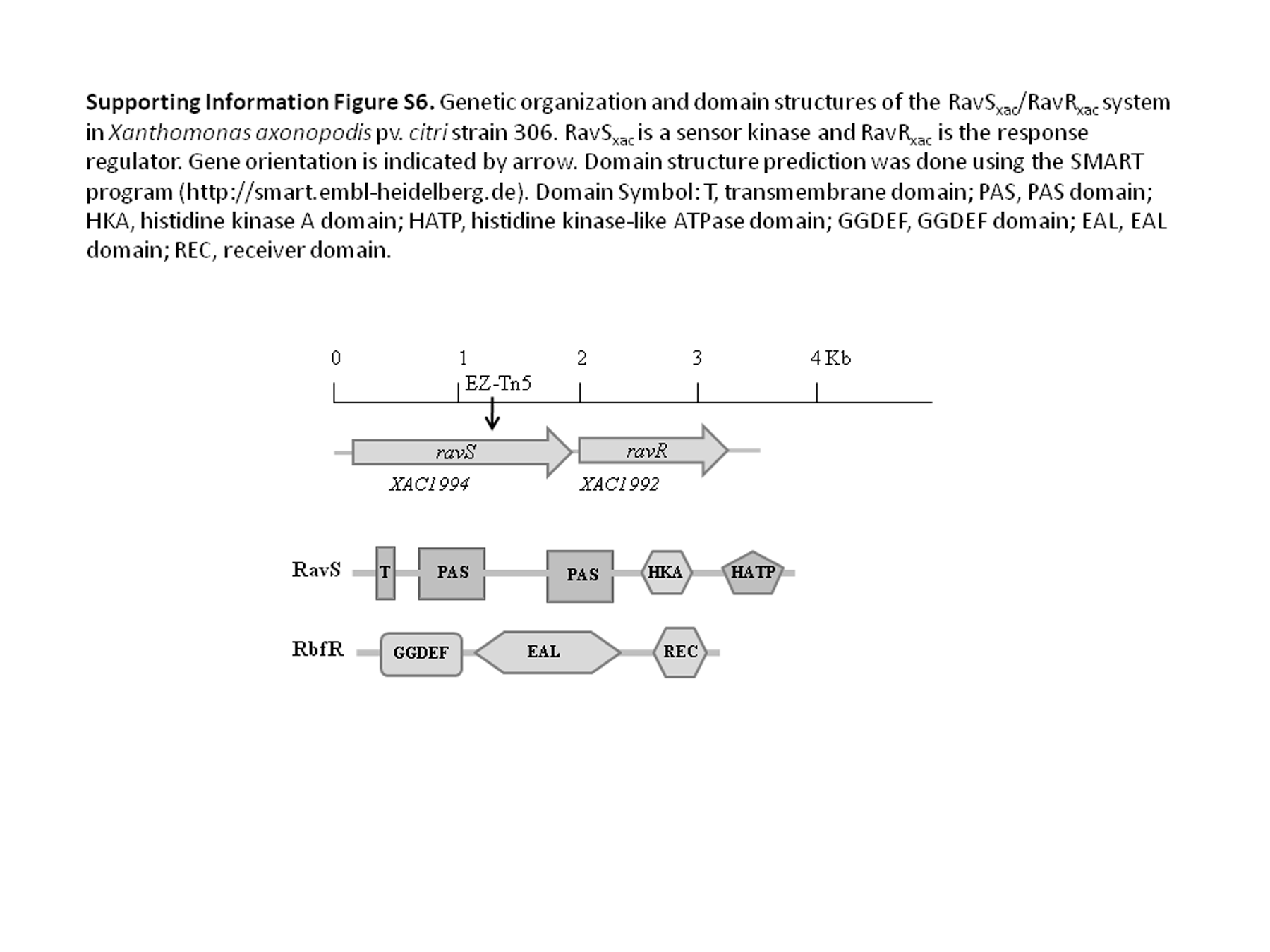

Supplement: Figure S6 — Genetic organization and domain structures of the RavSxac/RavRxac system in Xanthomonas axonopodis pv. citri strain 306. RavSxac is a sensor kinase and RavRxac is the response regulator. Gene orientation is indicated by arrow. Domain structure prediction was done using the SMART program (http://smart.embl-heidelberg.de). Domain Symbol: T, transmembrane domain; PAS, PAS domain; HKA, histidine kinase A domain; HATP, histidine kinase-like ATPase domain; GGDEF, GGDEF domain; EAL, EAL domain; REC, receiver domain. (TIF) [file pone.0021804.s006.tif]
