# Supplementary material for: Genome-Wide Mutagenesis of Xanthomonas axonopodis pv. citri Reveals Novel Genetic Determinants and Regulation Mechanisms of Biofilm Formation
Source: PLoS One. 2011 Jul 5;6(7):e21804. doi: 10.1371/journal.pone.0021804 (PMC3130047; doi:10.1371/journal.pone.0021804)
Supplement: Figure S3 — Genetic organization of biofilm-formation-related gene clusters in Xanthomonas axonopodis pv. citri strain 306. (a) Gum genes cluster, (b) LPS biosynthesis genes cluster, (c) EPS and LPS precursors biosynthesis gene cluster, (d) hemagglutinin coding genes cluster, (e) Chemotaxis/flagellum/motility genes cluster, and (f) pili gene cluster. Black triangles indicate insertion sites of the EZ-Tn5 transposon. (PDF) [file pone.0021804.s003.pdf]

Supporting Information Figure S3. Genetic organization of biofilm-formation-related gene clusters in *Xanthomonas axonopodis* pv. *citri* strain 306. (a) Gum genes cluster, (b) LPS biosynthesis genes cluster, (c) EPS and LPS precursors biosynthesis gene cluster, (d) hemagglutinin coding genes cluster, (e) Chemotaxis/flagellum/motility genes cluster, and (f) pili gene cluster. Black triangles indicate insertion sites of the EZ-Tn5 transposon.

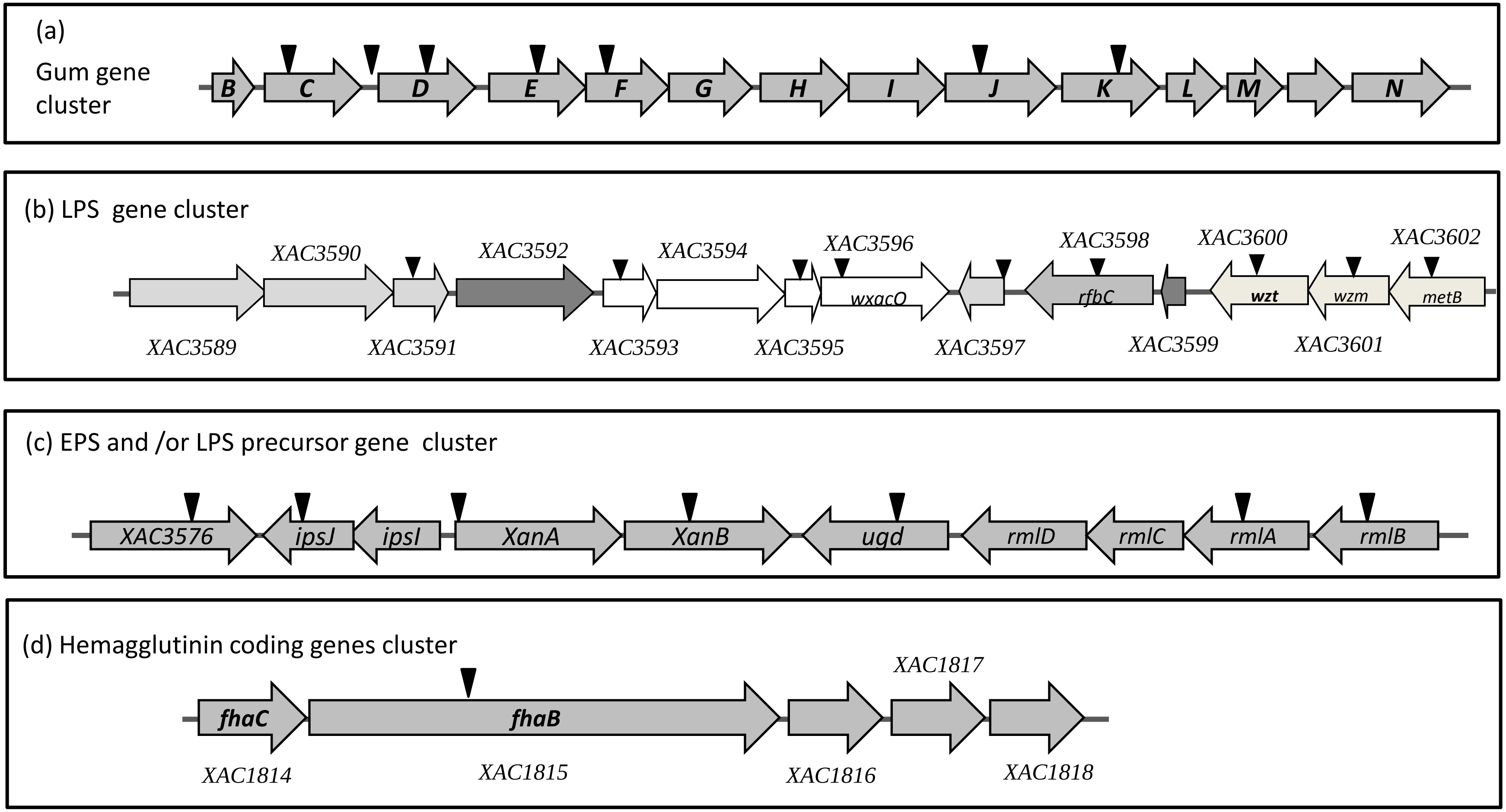

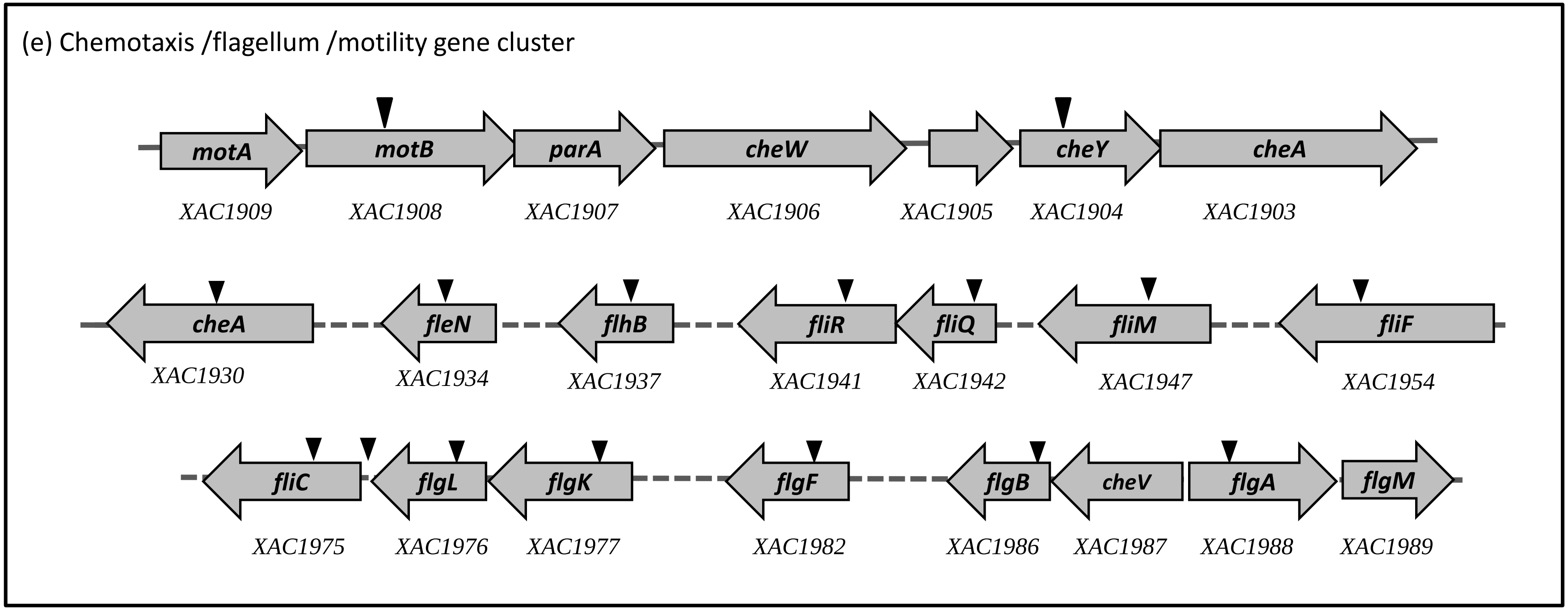
(e) Chemotaxis /flagellum /motility gene cluster
motA
motB
parA
cheW
cheY
cheA
XAC1909
XAC1908
XAC1907
XAC1906
XAC1905
XAC1904
XAC1903
cheA
fleN
flhB
fliR
fliQ
fliM
fliF
XAC1930
XAC1934
XAC1937
XAC1941
XAC1942
XAC1947
XAC1954
fliC
flgL
flgK
flgF
flgB
cheV
flgA
flgM
XAC1975
XAC1976
XAC1977
XAC1982
XAC1986
XAC1987
XAC1988
XAC1989

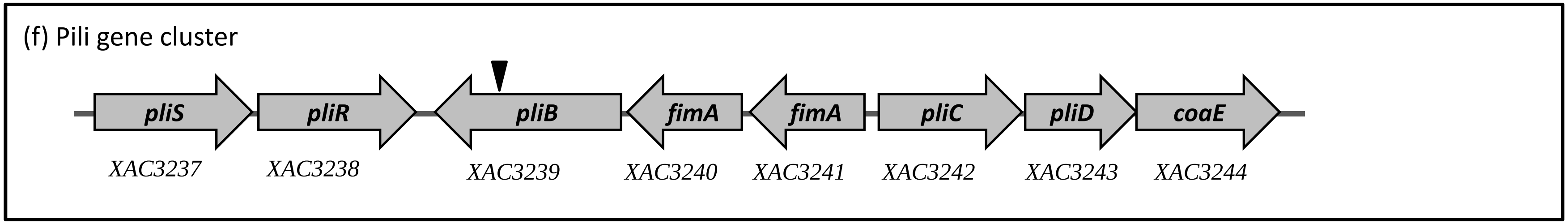
(f) Pili gene cluster
pliS
pliR
pliB
fimA
fimA
pliC
pliD
coaE
XAC3237
XAC3238
XAC3239
XAC3240
XAC3241
XAC3242
XAC3243
XAC3244
