# Supplementary material for: Genome-Wide Mutagenesis of Xanthomonas axonopodis pv. citri Reveals Novel Genetic Determinants and Regulation Mechanisms of Biofilm Formation
Source: PLoS One. 2011 Jul 5;6(7):e21804. doi: 10.1371/journal.pone.0021804 (PMC3130047; doi:10.1371/journal.pone.0021804)
Supplement: Figure S4 — Assays for EPS production (A) and cell motility (B) of biofilm-defective mutants of Xanthomonas axonopodis pv. citri strain 306. For the EPS assay, a modified ethanol deposit method was applied. For the motility test, bacterial strains were inoculated at a central point on NA plates (0.3% agar for swimming assays and 0.7% agar for swarming assays) and incubated at room temperature (approximately 23°C) for 7 days, after which colony diameters were measured. All experiments were repeated three times with three replicates. Averages and standard errors from one of three representative experiments with similar results are presented. Wild-type strain 306 was set as equal to 100%. Significance was tested by Student's t-test (* indicates significant difference from wild-type strain 306 at P<0.01). Wt306: wild type strain 306; 294E2(XAC0144/iroN): EZ-Tn5 insertion in XAC0144; 332D5(XAC0482/bdp1): EZ-Tn5 insertion in XAC0482; 283C5(XAC0483/clp); EZ-Tn5 insertion in XAC0483; 301B4(XAC0494/bdp18/rbfS): EZ-Tn5 insertion in XAC0494; 421F4(XAC0655/adk): EZ-Tn5 insertion in XAC0655; 285E8(XAC0721/bdp2): EZ-Tn5 insertion in XAC0721; 294E4(XAC0929/bdp19): EZ-Tn5 insertion in XAC0929; 270H4(XAC1469/bdp3): EZ-Tn5 insertion in XAC1469; 319B1(XAC1499/bdp20): EZ-Tn5 insertion in XAC1499; 411F5(XAC1509/bdp4): EZ-Tn5 insertion in XAC1509; 296G1 (XAC1778/bdp21): EZ-Tn5 insertion in XAC1778; 288D11(XAC1923/bdp5): EZ-Tn5 insertion in XAC1923; 212C9(XAC1994/ravS):EZ-Tn5 insertion in XAC1994; 290E7(XAC2018/bdp6): EZ-Tn5 insertion in XAC2018; 295H3(XAC2848/bdp7): EZ-Tn5 insertion in XAC2848; 291G5(XAC2670/bdp23): EZ-Tn5 insertion in XAC2670; 223G4(XAC3110/bdp24): EZ-Tn5 insertion in XAC3110; 292G4(XAC3364/bdp8): EZ-Tn5 insertion in XAC3364; 207A6(XAC3576/bdp9): EZ-Tn5 insertion in XAC3576; 291A9(XAC3591/bdp25): EZ-Tn5 insertion in XAC3591; 225E4(XAC3597/bdp11): EZ-Tn5 insertion in XAC3597; 409D8(XAC4024/bdp13): EZ-Tn5 insertion in XAC4024; 228F10(XAC4203/bdp14): EZ-Tn5 insertion in XAC4203; 302B1 [file pone.0021804.s004.pdf]

Supporting Information Figure S4. Assays for EPS production (A) and cell motility (B) of biofilm-defective mutants of *Xanthomonas axonopodis* pv. *citri* strain 306. For the EPS assay, a modified ethanol deposit method was applied. For the motility test, bacterial strains were inoculated at a central point on NA plates (0.3% agar for swimming assays and 0.7% agar for swarming assays) and incubated at room temperature (approximately 23 C) for 7 days, after which colony diameters were measured. All experiments were repeated three times with three replicates. Averages and standard errors from one of three representative experiments with similar results are presented. Wild-type strain 306 was set as equal to 100%. Significance was tested by Student's *t*-test (* indicates significant difference from wild-type strain 306 at $P < 0.01$). Wt306: wild type strain 306; 294E2(XAC0144/iroN): EZ-Tn5 insertion in *XAC0144*; 332D5(XAC0482/bdp1): EZ-Tn5 insertion in *XAC0482;* 283C5(XAC0483/clp); EZ-Tn5 insertion in *XAC0483;* 301B4(XAC0494/bdp18/rbfS): EZ-Tn5 insertion in *XAC0494*; 421F4(XAC0655/adk): EZ-Tn5 insertion in *XAC0655*; 285E8(XAC0721/bdp2): EZ-Tn5 insertion in *XAC0721*; 294E4(XAC0929/bdp19): EZ-Tn5 insertion in *XAC0929*; 270H4(XAC1469/bdp3): EZ-Tn5 insertion in *XAC1469*; 319B1(XAC1499/bdp20): EZ-Tn5 insertion in *XAC1499*; 411F5(XAC1509/bdp4): EZ-Tn5 insertion in *XAC1509*; 296G1 (XAC1778/bdp21): EZ-Tn5 insertion in *XAC1778*; 288D11(XAC1923/bdp5): EZ-Tn5 insertion in *XAC1923*; 212C9(XAC1994/ravS):EZ-Tn5 insertion in *XAC1994*; 290E7(XAC2018/bdp6): EZ-Tn5 insertion in *XAC2018*; 295H3(XAC2848/bdp7): EZ-Tn5 insertion in *XAC2848*; 291G5(XAC2670/bdp23): EZ-Tn5 insertion in *XAC2670*; 223G4(XAC3110/bdp24): EZ-Tn5 insertion in *XAC3110*; 292G4(XAC3364/bdp8): EZ-Tn5 insertion in *XAC3364*; 207A6(XAC3576/bdp9): EZ-Tn5 insertion in *XAC3576*; 291A9(XAC3591/bdp25): EZ-Tn5 insertion in *XAC3591*; 225E4(XAC3597/bdp11): EZ-Tn5 insertion in *XAC3597*; 409D8(XAC4024/bdp13): EZ-Tn5 insertion in *XAC4024*; 228F10(XAC4203/bdp14): EZ-Tn5 insertion in *XAC4203*; 302B11(XAC4249/xynA): EZ-Tn5 insertion in *XAC4249*; 295D8 (XAC4264/bdp15): EZ-Tn5 insertion in *XAC4264*; 347D10(XAC4344/vacJ): EZ-Tn5 insertion in *XAC4344*; 288C6(XACa0007/bdp16): EZ-Tn5 insertion in *XACa0007*; 423H3(XACb0001/bdp17): EZ-Tn5 insertion in *XACb0001;* 419F10(XACb0050/bdp28): EZ-Tn5 insertion in *XACb0050;* 276B8(XAC1975/fliC): EZ-Tn5 insertion in *XAC1975/fliC,* and 257H6(XAC2583/gumD): EZ-Tn5 insertion in *XAC2583/gumD.*

(A)

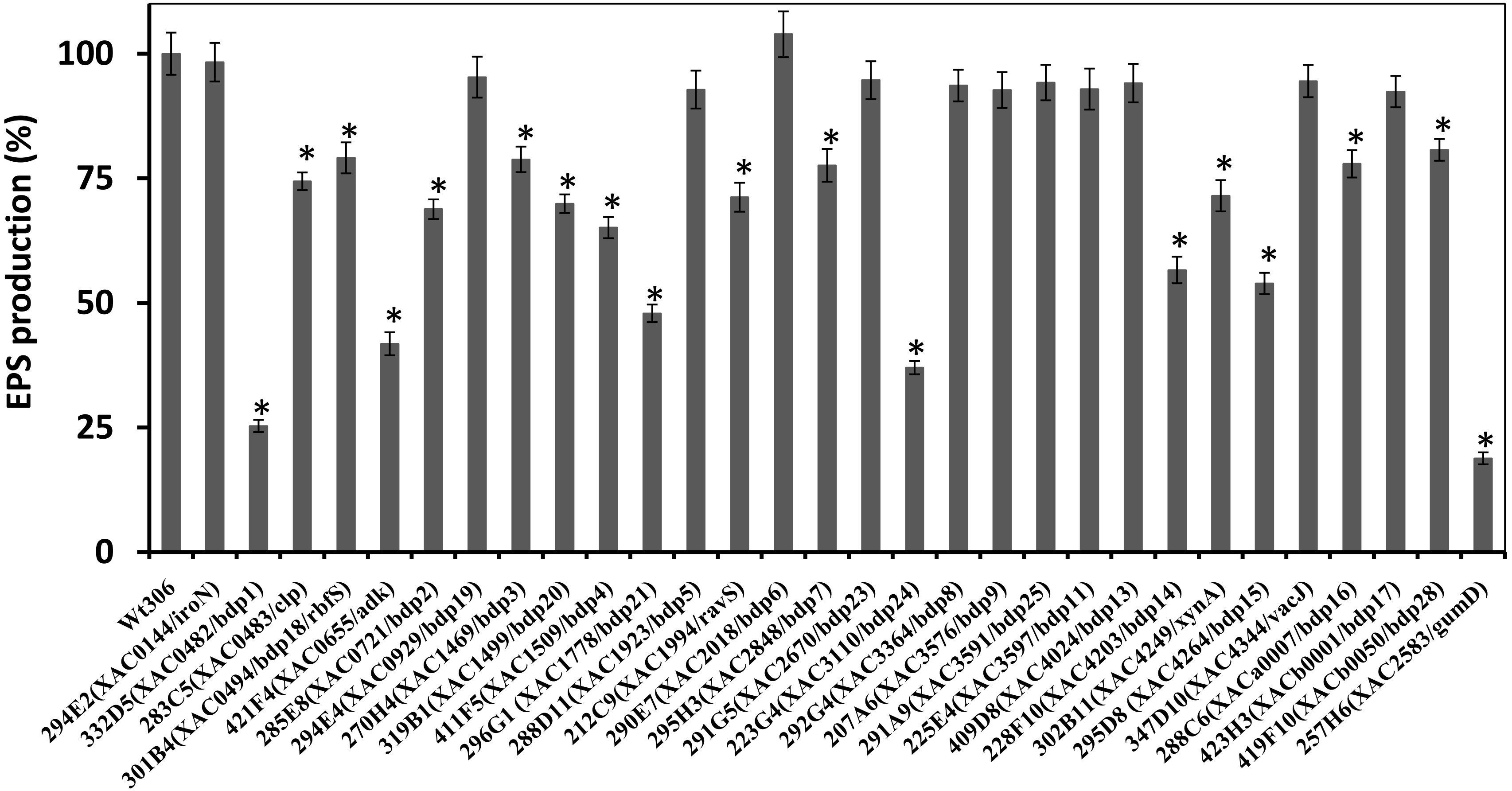

EPS production (%)
100
75
50
25
0
Wt306
294E2(XAC0144/iroN)
332D5(XAC0482/bdp1)
283C5(XAC0483/clp)
301B4(XAC0494/bdp18/rbfS)
421F4(XAC0655/adk)
285E8(XAC0721/bdp2)
294E4(XAC0929/bdp19)
270H4(XAC1469/bdp3)
319B1(XAC1499/bdp20)
411F5(XAC1509/bdp4)
296G1 (XAC1778/bdp21)
288D11(XAC1923/bdp5)
212C9(XAC1994/ravS)
290E7(XAC2018/bdp6)
295H3(XAC2848/bdp7)
291G5(XAC2670/bdp23)
223G4(XAC3110/bdp24)
292G4(XAC3364/bdp8)
207A6(XAC3576/bdp9)
291A9(XAC3591/bdp25)
225E4(XAC3597/bdp11)
409D8(XAC4024/bdp13)
228F10(XAC4203/bdp14)
302B11(XAC4249/xynA)
295D8 (XAC4264/bdp15)
347D10(XAC4344/vacJ)
288C6(XACa0007/bdp16)
423H3(XACb0001/bdp17)
419F10(XACb0050/bdp28)
257H6(XAC2583/gumD)

(B)

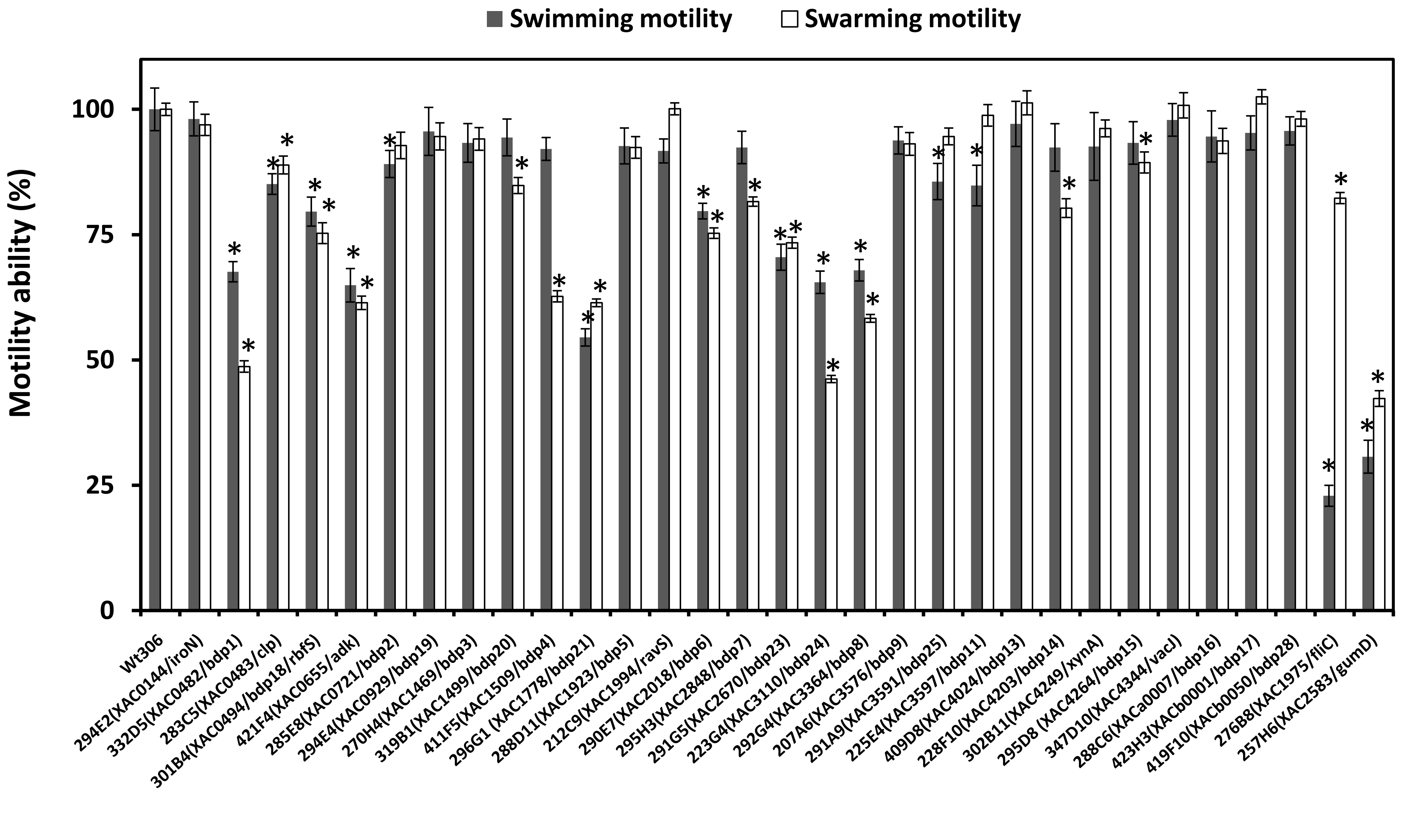

Swimming motility
Swarming motility
Motility ability (%)
100
75
50
25
0
Wt306
294E2(XAC0144/iroN)
332D5(XAC0482/bdp1)
283C5(XAC0483/clp)
301B4(XAC0494/bdp18/rbfS)
421F4(XAC0655/adk)
285E8(XAC0721/bdp2)
294E4(XAC0929/bdp19)
270H4(XAC1469/bdp3)
319B1(XAC1499/bdp20)
411F5(XAC1509/bdp4)
296G1 (XAC1778/bdp21)
288D11(XAC1923/bdp5)
212C9(XAC1994/ravS)
290E7(XAC2018/bdp6)
295H3(XAC2848/bdp7)
291G5(XAC2670/bdp23)
223G4(XAC3110/bdp24)
292G4(XAC3364/bdp8)
207A6(XAC3576/bdp9)
291A9(XAC3591/bdp25)
225E4(XAC3597/bdp11)
409D8(XAC4024/bdp13)
228F10(XAC4203/bdp14)
302B11(XAC4249/xynA)
295D8 (XAC4264/bdp15)
347D10(XAC4344/vacJ)
288C6(XACa0007/bdp16)
423H3(XACb0001/bdp17)
419F10(XACb0050/bdp28)
276B8(XAC1975/fliC)
257H6(XAC2583/gumD)
