# Supplementary material for: Genome-Wide Mutagenesis of Xanthomonas axonopodis pv. citri Reveals Novel Genetic Determinants and Regulation Mechanisms of Biofilm Formation
Source: PLoS One. 2011 Jul 5;6(7):e21804. doi: 10.1371/journal.pone.0021804 (PMC3130047; doi:10.1371/journal.pone.0021804)
Supplement: Table S1 — Biofilm related genes identified from Xanthomonas axonopodis pv. citri strain 306 in this study. (DOC) [file pone.0021804.s007.doc]

**Supporting Information Table S1. Biofilm related genes identified from *Xanthomonas axonopodis* pv. *citri* strain 306 in this study.**

| Gene category(n^a^) | Locus of  EZ-Tn5 tagged ^b^ | Gene name(s) | Gene product or domain similarity ^c^ | Polar effect possible^d^ | Mutant strains |
| --- | --- | --- | --- | --- | --- |
| **Metabolism (23)** |  |  |  |  |  |
| Carbohydrate metabolism (3) | *XAC3579* | *xanA* | phosphoglucomutase | no | 362C10 |
|  | *XAC3580* | *xanB* | GDP-mannose pyrophosphorylase | no | 287E3 |
|  | *XAC3581* | *ugd* | UDP-glucose dehydrogenase | no | 293G1 |
| Glycan biosynthesis and metabolism (6) | *XAC1094* | *opsX* | saccharide biosynthesis regulatory protein | no | 407B9 |
|  | *XAC2576* | *gumK* | GumK protein | yes | 246F8 |
|  | *XAC2577* | *gumJ* | GumJ protein | no | 244E7 |
|  | *XAC2581* | *gumF* | GumF protein | yes | 289G3 |
|  | *XAC2582* | *gumE* | GumE protein | yes | 411A5 |
|  | *XAC2584* | *gumC* | GumC protein | no | 413A7 |
| Amino acid metabolism (6) | *XAC0804* | *sahH* | S-adenosyl-L-homocysteine hydrolase | no | 291D4 |
|  | *XAC1130* | *trpE* | anthranilate synthase component I | no | 296C6 |
|  | *XAC1533* | *ldp* | dihydrolipoamide dehydrogenase | no | 245B4 |
|  | *XAC3302* | *thiG* | thiazole synthase | no | 216E9 |
|  | *XAC3577* | *ipsJ* | IpsJ protein/3-oxoacid CoA transferase beta subunit | no | 411B4 |
|  | *XAC3602* | *metB* | cystathionine gamma-lyase-like protein | yes | 225B5 |
| Cofactors and vitamins metabolism (3) | *XAC0388* | *bioB* | biotin synthase | no | 295C11 |
|  | *XAC0927* | *ilvE* | branched-chain amino acid aminotransferase | no | 410D11 |
|  | *XAC3415* | *thiE* | thiamine-phosphate pyrophosphorylase | yes | 225C2 |
| Biosynthesis of other secondary metabolites (2) | *XAC3584* | *rmlA* | glucose-1-phosphate thymidylyltransferase | yes | 299E12 |
|  | *XAC3585* | *rmlB* | dTDP-glucose 4,6-dehydratase | yes | 227E11 |
| Xenobiotics biodegradation and metabolism (1) | *XAC1879* | *rpfF* | RpfF protein/ enoyl-CoA hydratase | no | 219H10 |
| Nucleotide metabolism (1) | *XAC0655* | *adk_Xac_* | sugar kinase/ adenosine kinase | no | 421F4 |
| Energy metabolism (1) | *XAC2692* | *nuoM* | NADH dehydrogenase subunit M | yes | 254F9 |
| **Genetic information processing (4)** |  |  |  |  |  |
| Replication and repair (2) | *XAC0660* | *mrdB* | rod shape-determining protein | no | 242E11 |
|  | *XAC4074* | *nrdF* | ribonucleotide-diphosphate reductase subunit beta | no | 282D10 |
| Transcription (2) | *XAC0483* | *clp* | cAMP-regulatory protein | no | 283C5 |
|  | *XAC1969* | *rpoN* | RNA polymerase sigma-54 factor | no | 411F11 |
| **Signal transduction (2)** |  |  |  |  |  |
|  | *XAC1878* | *rpfC* | RpfC protein /sensor histidine kinase | no | 228D8 |
|  | *XAC1994* | *ravS_Xac_* | HrpX -like protein/two-component system sensor protein | yes | 212C9 |

**Supporting Information Table S1. continued.**

| Gene category(n^a^) | Locus of  EZ-Tn5 tagged^b^ | | Gene  name(s) | Gene product or domain similarity^c^ | | Polar effect  possible^d^ | | Mutant  strains |
| --- | --- | --- | --- | --- | --- | --- | --- | --- |
| **Cellular Processes (19)** |  |  | |  | |  | |  |
| Bacterial chemotaxis (4) | *XAC0611* | | *tsr* | | chemotaxis protein | | no | 214G11 |
|  | *XAC1746* | | *mcpA* | | chemotaxis protein | | no | 304A1 |
|  | *XAC1904* | | *cheY* | | chemotaxis response regulator | | yes | 253C5 |
|  | *XAC1930* | | *cheA* | | chemotaxis related protein | | no | 281B3 |
| Flagellar assembly (11) | *XAC1934* | | *fleN* | | flagellar biosynthesis switch protein | | yes | 235C4 |
|  | *XAC1937* | | *flhB* | | flagellar biosynthesis protein | | yes | 282D2 |
|  | *XAC1941* | | *fliR* | | flagellar biosynthesis protein | | no | 262G8 |
|  | *XAC1942* | | *fliQ* | | flagellar biosynthesis protein | | no | 305E11 |
|  | *XAC1947* | | *fliM* | | flagellar motor switch protein | | no | 290E8 |
|  | *XAC1954* | | *fliF* | | flagellar MS-ring protein | | yes | 296F10 |
|  | *XAC1976* | | *flgL* | | flagellar hook-associated protein | | no | 335G4 |
|  | *XAC1977* | | *flgK* | | flagellar hook-associated protein | | yes | 299H12 |
|  | *XAC1982* | | *flgF* | | flagellar basal body rod protein | | no | 239A1 |
|  | *XAC1986* | | *flgB* | | flagellar basal body rod protein | | no | 278G3 |
|  | *XAC1988* | | *flgA* | | flagellar basal body P-ring biosynthesis protein | | yes | 216D4 |
| Cell motility (2) | *XAC1908* | | *motB* | | flagellar motor protein | | yes | 218B3 |
|  | *XAC3694* | | *motB* | | flagellar motor protein | | no | 416D1 |
| Type IV pilus assembly (1) | *XAC3239* | | *pilB* | | pilus biogenesis protein | | no | 426A6 |
| Cytoskeleton proteins (1) | *XAC1225* | | *minD* | | septum site-determining protein | | yes | 308B5 |
| **Membrane transport (4)** | *XAC1017* | | *sbp* | | ABC transporter sulfate binding protein | | yes | 419B5 |
|  | *XAC1459* | | *msbA* | | ABC transporter ATP-binding protein | | no | 376B4 |
|  | *XAC3600* | | *wzt* | | ABC transporter ATP-binding protein | | no | 337E2 |
|  | *XAC3601* | | *wzm* | | ABC transporter permease | | yes | 335D7 |
| **Hypothetical genes (17)** | *XAC0482* | | *bdp1* | | hypothetical protein, similar to phosphatase | | no | 332D5 |
|  | *XAC0721* | | *bdp2* | | hypothetical protein, predicted endonuclease /exonuclease /phosphatase family | | no | 285E8 |
|  | *XAC1469* | | *bdp3* | | hypothetical protein, similar to TatD related DNase | | no | 270H4 |
|  | *XAC1509* | | *bdp4* | | hypothetical protein | | no | 411F5 |
|  | *XAC1923* | | *bdp5* | | hypothetical protein | | no | 288D11 |
|  | *XAC2018* | | *bdp6* | | hypothetical protein, predicted transcriptional regulator containing XRE-like HTH domain | | no | 290E7 |
|  | *XAC2848* | | *bdp7* | | hypothetical protein, similar to lipocalin-like protein | | no | 295H3 |
|  | *XAC3364* | | *bdp8* | | hypothetical protein, similar to acetyl-CoA hydrolase | | no | 292G4 |
|  | *XAC3576* | | *bdp9* | | hypothetical protein, putative carbohydrate biosynthesis protein contains Pfam_Rgpf domain | | no | 207A6 |
|  | *XAC3595* | | *bdp10* | | hypothetical protein, similar to transmembrane GtrA-like cell surface polysaccharide biosynthesis protein | | yes | 246F9 |
|  | *XAC3597* | | *bdp11* | | hypothetical protein, similar to Phytanoyl-CoA dioxygenase, Phyh superfamily | | no | 225E4 |
|  | *XAC3743* | | *bdp12* | | hypothetical protein, similar to carbohydrate-binding enzyme, Glycosyl hydrolase family 1 | | yes | 289A1 |
|  | *XAC4024* | | *bdp13* | | hypothetical protein, similar to outer membrane protein | | no | 409D8 |
|  | *XAC4203* | | *bdp14* | | hypothetical protein, similar to putative pathogenicity protein | | no | 228F10 |
|  | *XAC4264* | | *bdp15* | | hypothetical protein, similar to sucrose isomerase | | no | 295D8 |
|  | *XACa0007* | | *bdp16* | | hypothetical protein, similar to protein kinase C | | no | 288C6 |
|  | *XACb0001* | | *bdp17* | | hypothetical protein, similar to Radical SAM domain protein | | no | 423H3 |

**Supporting Information Table S1. continued.**

| Gene category(n^a^) | Locus of  EZ-Tn5 tagged^b^ | Gene  name(s) | | Gene product or domain similarity ^c^ | | | Polar effect  possible^d^ | | Mutant  strains |
| --- | --- | --- | --- | --- | --- | --- | --- | --- | --- |
| **Other not well characterized (16)** |  |  | |  | | |  | |  |
|  | *XAC0144* | *iroN* | | TonB-dependent outer membrane receptor | | | no | | 294E2 |
|  | *XAC0494* | *bdp18/rbfS* | | two-component system sensor protein | | | no | | 301B4 |
|  | *XAC0929* | *bdp19* | | extracellular protease | | | no | | 294E4 |
|  | *XAC1499* | *bdp20* | | transcriptional regulator, XRE family | | | no | | 319B1 |
|  | *XAC1778* | *bdp21* | | sensor kinase, MASE1-containing protein, similar to sugar transporter component | | | no | | 296G1 |
|  | *XAC2293* | *bdp22* | | dehydratase protein | | | yes | | 293C3 |
|  | *XAC2294* | *rbp303* | | lipopolysaccharide core biosynthesis protein | | | yes | | 424C1 |
|  | *XAC2670* | *bdp23* | | alginate biosynthesis protein, similar to two-component system sensor protein | | | no | | 291G5 |
|  | *XAC3110* | *bdp24* | | glycosyltransferase, glycosyl transferase family 2 | | | no | | 223G4 |
|  | *XAC3217* | *rluD* | | ribosomal large subunit pseudouridine synthase D | | | yes | | 233C5 |
|  | *XAC3591* | *bdp25* | | short chain dehydrogenase | | | no | | 291A9 |
|  | *XAC3593* | *bdp26* | | NAD dependent epimerase/dehydratase/dehydrogenase | | | yes | | 267F7 |
|  | *XAC3960* | *bdp27* | | oxidoreductase | | | yes | | 296E3 |
|  | *XAC4249* | *xynA* | | endo-1,4-beta-xylanase | | | no | | 302B11 |
|  | *XAC4344* | *vacJ* | | lipoprotein | | | no | | 347D10 |
|  | *XACb0050* | *bdp28* | | ISxac2 transposase | | | no | | 419F10 |
| **Intergenic region** **(4)** | |  | |  | | |  | |  |
|  | *XAC1975/*  *XAC1976* | *fliC/flgL* | | flagellin /flagellar hook-associated protein | | |  | | 241G4 |
|  | *XAC0226 / XAC0227* |  | | two-component system regulatory protein/ hypothetical protein XAC0227 | | |  | | 289F6 |
|  | *XAC2369 / XAC2370* |  | | general stress protein XAC2369/hypothetical protein XAC2370 | | |  | | 407D9 |
|  | *XAC2583/ XAC2584* | *gumD/ gumC* | | GumD protein/ GumC protein | | |  | | 269D7 |
| **Previously reported genes (7)** | | |  | |  |  | |  | |
|  | *XAC1975* | *fliC* | | flagellin | | | no | | 276B8 |
|  | *XAC1815* | *fhaB* | | filamentous haemagglutinin (adhesin) | | | no | | 304E5 |
|  | *XAC2292* | *galU* | | UTP-glucose-1-phosphate uridylyltransferase | | | no | | 292A2 |
|  | *XAC2583* | *gumD* | | GumD protein | | | no | | 257H6 |
|  | *XAC3250* | *colR* | | two-component system regulatory protein | | | no | | 289H9 |
|  | *XAC3596* | *wxacO* | | outer membrane protein | | | no | | 302F9 |
|  | *XAC3598* | *rfbC* | | truncated O-antigen biosynthesis protein | | | no | | 240H1 |

*^a^ n*, number of genes involved in that pathway, as suggested by the KEGG pathway.

*^b^* Open reading frame numbering (ordered sequence tag) from the strain 306 genome.

*^c^* Based on BLASTP search and SMART analyses.

*^d^* Polar effect was predicted by operon organization of the inserted genes relative to t ranscription sense of EZ-Tn5.
