# Supplementary material for: Genome-Wide Mutagenesis of Xanthomonas axonopodis pv. citri Reveals Novel Genetic Determinants and Regulation Mechanisms of Biofilm Formation
Source: PLoS One. 2011 Jul 5;6(7):e21804. doi: 10.1371/journal.pone.0021804 (PMC3130047; doi:10.1371/journal.pone.0021804)
Supplement: Table S2 — Bacterial strains and plasmids used in this studya. (DOC) [file pone.0021804.s008.doc]

**Supporting Information Table S2. Bacterial strains and plasmids used in this study^a^.**

| Strains and plasmids | Characteristics | Reference or source |
| --- | --- | --- |
| Strains |  |  |
| *E. coli* |  |  |
| DH5α | F^–^ *recA1 endA1 hsdR17 supE44 thi-1 gyrA96 relA1* Δ (*argE-lacZYA*)*169*ϕ80*lazA* Δ M15 | [1] |
| JM109 | *rec*A1, *end*A1, *gyr*A96, *thi*, *hsd*R17 (rK–,mK+), *rel*A1, *sup*E44, Δ(*lac*-*pro*AB), [F´, *tra*D36, *pro*AB, *lac*IqZΔM15] | Promega |
| HB101 | F^–^ *supE44, hsdS20(rB^-^ mB^-^), recA13, ara-14, proA2, lacY1, galK2, rpsL20, xyl-5, mtl-1, leuB6, thi* | [2] |
| *X. axonopodis* pv. *citri* |  |  |
| 306 | wild type, pathogenic to citrus, Rf^r^ | [3] |
| 332D5 | *XAC0482*::EZ-Tn5 derivative of strain 306, Rf^r^, Km^r^ | This study |
| 301B4 | *rbfS (XAC0494)*::EZ-Tn5 derivative of strain 306, Rf^r^, Km^r^ | This study |
| Plasmids |  |  |
| pGEM®-T Easy | PCR cloning and sequencing vector, *lacZ*’, Ap^r^ | Promega |
| pRK2013 | ColE1 Tra^+^, conjugation helper plasmid, Km^r^ | [4] |
| pUFR053 | IncW Mob^+^*mob*(P) *lac*Zα^+^ Par^+^, Cm^r^, Gm^r^, shuttle vector | [5] |
| pUF-0482 | A 1240 bp *Bam*HI-*Hind*III fragment containing wild-type *XAC0482* gene ligated into pUFR053, Cm^r^, Gm^r^ | This study |
| pUF-rbfS | A 2450 bp *Bam*HI-*Hind*III fragment containing wild-type *rbfS (XAC0494)* gene ligated into pUFR053, Cm^r^, Gm^r^ | This study |

*^a^* Ap^r^,Cm^r^, Gm^r^, Km^r^, and Rf^r^ indicate resistance to ampicillin, chloromycetin, gentamicin, kanamycin, and rifamycin, respectively.

**Literature cited in Supporting Information Table S2**

1. Hanahan F **(**1983) Studies on transformation of *Escherichia coli* with plasmids. J Mol Biol 166: 557-580.
2. Boyer H, Roulland-Dussoix D (1969) A complementation analysis of the restriction and modification of DNA in *Escherichia coli*. J Mol Biol 41: 459-472.
3. Rybak M, Minsavage GV, Stall E, Jones JB (2009) Identification of *Xanthomonas citri* subsp. *citri* host specificity genes in a heterologous expression host. Mol Plant Pathol 10:249–262.
4. Figurski DH, Helinski DR **(**1979) Replication of an origin-containing derivative of plasmid RK2 dependent on a plasmid function provided *in trans*. Proc Natl Acad Sci USA 76: 1648-1652.
5. El Yacoubi B, Brunings A, Yuan Q, Shankar S, Gabriel D (2007) *In planta* horizontal transfer of a major pathogenicity effector gene. Appl Environ Microbiol 73: 1612-1621.
