# Supplementary material for: Genome-Wide Mutagenesis of Xanthomonas axonopodis pv. citri Reveals Novel Genetic Determinants and Regulation Mechanisms of Biofilm Formation
Source: PLoS One. 2011 Jul 5;6(7):e21804. doi: 10.1371/journal.pone.0021804 (PMC3130047; doi:10.1371/journal.pone.0021804)
Supplement: Table S3 — Primers used in this study. (DOC) [file pone.0021804.s009.doc]

**Supporting Information Table S3. Primers used in this study.**

| Primer | Sequence (5’ → 3’)^α^ |
| --- | --- |
| Primers for cloning and sequencing of EZ-Tn5-flanking sequences |  |
| Inv-1 | ATGGCTCATAACACCCCTTGTATTA |
| Inv-2 | GAACTTTTGCTGAGTTGAAGGATCA |
| KAN-2 FP-1 | ACCTACAACAAAGCTCTCATCAACC |
| KAN-2 RP-1 | CTACCCTGTGGAACACCTACATCT |
| Primers for amplification of DNA fragment of Kanamycin resistance gene used as probe |  |
| Kan-F1 | ATTCAACGGGAAACGTCTTG |
| Kan-R1 | CGAGCATCAAATGAAACTGC |
| Primers for amplifying *XAC0482* used in complementation |  |
| C82-F (*Bam*HI) | ATCGATggatccGCATCCTTTGATCGTCTCC |
| C82-R (*Hin*dIII) | ACTTAGaagcttGCGTCACGATCCTGAAGATT |
| Primers for amplifying *XAC0494* used in complementation |  |
| C94-F (*Bam*HI) | TCTACTggatccGCAAGGAACTGGATTCGAC |
| C94-R (*Hin*dIII) | ATCTGTaagattGAAGGCGTTGAGCATCAGAG |

^α^ lowercase nucleotides are not exact matches to the sequence and were introduced to add restriction enzyme site.
